# Association of Medicaid Expansion With 5-Year Changes in Hypertension and Diabetes Outcomes at Federally Qualified Health Centers

**DOI:** 10.1001/jamahealthforum.2021.2375

**Published:** 2021-09-10

**Authors:** Megan B. Cole, June-Ho Kim, Timothy W. Levengood, Amal N. Trivedi

**Affiliations:** 1 Department of Health Law, Policy, and Management, Boston University School of Public Health, Boston, Massachusetts; 2 Ariadne Labs, Brigham and Women’s Hospital, Harvard T.H. Chan School of Public Health, Boston, Massachusetts; 3 Division of General Internal Medicine & Primary Care, Brigham and Women’s Hospital, Boston, Massachusetts; 4 Department of Health Services, Policy, and Practice, Brown University School of Public Health, Providence, Rhode Island; 5 Center of Innovation for Long-term Services and Supports, Providence VA Medical Center, Providence, Rhode Island

## Abstract

**Importance:**

State decisions to expand Medicaid eligibility were particularly consequential for federally qualified health centers (FQHCs), which serve 30 million low-income patients across the US. The longer-term association of Medicaid expansion with health outcomes at FQHCs is unknown.

**Objective:**

To assess the 5-year association of Medicaid expansion with uninsurance rates and hypertension and diabetes outcome measures by race and ethnicity in a nationally representative population of FQHCs.

**Design, Setting, and Participants:**

Using a difference-in-differences analysis of a retrospective cohort from the universe of US FQHCs, changes in uninsurance rates and intermediate health outcomes for hypertension and diabetes by race and ethnicity were compared between Medicaid expansion and nonexpansion states before (2012-2013) vs after (2014-2018) expansion. Data were analyzed from September 2020 to March 2021.

**Exposures:**

Location in a state that expanded Medicaid eligibility as of 2014.

**Main Outcomes and Measures:**

Rates of uninsurance, the proportion of patients with hypertension with a blood pressure less than 140/90 mm Hg, and the proportion of patients with diabetes with glycosylated hemoglobin levels of 9% or less, as stratified by race and ethnicity.

**Results:**

Of the patients at 578 expansion-state FQHCs (serving 13.0 million patients per year) and 368 nonexpansion-state FQHCs (serving 6.0 million patients per year) in our study sample, 64.4% were age 18 to 64 years, 57.4% were women, 18.9% were non-Hispanic Black, and 27.3% were Hispanic. Following expansion, FQHCs in Medicaid expansion states experienced a 9.24 percentage point (PP) (95% CI, 7.94-10.54) decline in rates of uninsurance over the pooled 5-year expansion period compared with nonexpansion-state FQHCs. Across this 5-year period, expansion was associated with a 1.61-PP (95% CI, 0.58-2.64) comparative improvement in hypertension control and a 1.84-PP (95% CI, 0.71-2.98) comparative improvement in glucose control. Stratified results suggest that improvements were consistently observed in Black and Hispanic populations. The magnitude of change tended to increase with implementation time. For instance, by year 5, expansion was associated with a 3.38-PP (95% CI, 0.80-5.96) comparative improvement in hypertension control and a 3.88-PP (95% CI, 0.86-6.90) comparative improvement in glucose control among Black populations.

**Conclusions and Relevance:**

In this nationally representative cohort study, Medicaid expansion was associated with sustained increases in insurance coverage and improvements in chronic disease outcome measures at FQHCs after 5 years overall and among Black and Hispanic populations. States considering Medicaid expansion may benefit from improved longer-run health measures for underserved patients with chronic conditions.

## Key Points

**Question:**

What has been the 5-year association of Medicaid expansion with uninsurance rates, hypertension and diabetes outcomes, and racial and ethnic differences in outcomes in a national sample of federally qualified health centers (FQHCs)?

**Findings:**

In this cohort study using a difference-in-differences analysis of 946 FQHCs that serve 18.9 million patients per year, Medicaid expansion-state FQHCs experienced improved blood pressure and glucose control measures over 5 years overall and for Black and Hispanic patients compared with FQHCs in nonexpansion states. Expansion was also associated with sustained reductions in uninsurance at FQHCs.

**Meaning:**

The findings of this cohort study suggest that Medicaid expansion was associated with better 5-year health performance outcomes for FQHCs, which may be important for states that are considering Medicaid expansion.

## Teaser

This cohort study examines the 5-year association of Medicaid expansion with uninsurance rates and hypertension and diabetes outcome measures by race and ethnicity in federally qualified health centers.

## Introduction

Federally qualified health centers (FQHCs) provide comprehensive primary care services to nearly 30 million low-income patients across the US without concern for ability to pay.^1,2^ Federally qualified health centers serve 1 in 5 Medicaid enrollees, 1 in 5 rural residents, and 1 in 3 persons with an income lower than the federal poverty level (FPL) in the US.^3^ In 2013, before implementation of Medicaid expansion under the US Affordable Care Act (ACA), 44% of adults who were utilizing FQHCs did not have insurance, 92% had incomes less than 200% of the FPL, and 65% were members of racial and ethnic minority groups.^2,4^

As of January 2014, 25 states and Washington, DC had expanded Medicaid eligibility^5^ to nonelderly adults with incomes up to 138% of the FPL, which was made optional to states as a result of a 2012 US Supreme Court ruling. Since then, 13 additional states have adopted Medicaid expansion, while 12 remaining states have not expanded eligibility as of 2020.^5^ State decisions to expand Medicaid were particularly consequential for FQHCs and the patients they serve, as those receiving care at FQHCs in expansion states experienced large gains in insurance coverage, whereas high rates of uninsurance in nonexpansion states persisted.^4,6,7^ In addition, Medicaid expansion may improve the financial revenues of FQHCs,^8,9^ which may allow FQHCs to expand capacity and improve quality of care not just for the newly insured, but for all FQHC patients.

Prior evidence has found that in the short term, Medicaid expansion was associated with improved quality of care at FQHCs and increases in FQHC service capacity,^4,8,10^ particularly for FQHCs located in rural areas.^6^ However, limited evidence exists about the longer-term associations of Medicaid expansion with FQHCs nationally. It may take years for expanded insurance coverage to yield hypothesized improvements in clinical outcome measures, as opposed to measures of care processes.

Evaluating changes in hypertension and diabetes control following expanded Medicaid coverage is especially important because uninsured and underinsured persons with these chronic conditions often forgo effective treatment that can reduce cardiovascular risk, mortality, and other complications.^11,12,13,14,15^ Moreover, understanding changes across racial and ethnic groups is important because hypertension and diabetes disproportionately affect some racial and ethnic minority groups,^16^ with persistent disparities in outcomes such as blood pressure and glucose control.^17^ Medicaid expansion provided an opportunity to potentially mitigate disparities in these health outcomes.

As such, our objective was to examine 5-year changes in uninsurance, blood pressure control, and glucose control following Medicaid expansion in a nationally representative population of all FQHCs. Specifically, we evaluate changes in FQHC-level performance, as opposed to changes in individual-level patient outcomes.

## Methods

### Data Source and Study Population

We used the Health Resources and Services Administration (HRSA) 2012 to 2018 Uniform Data System (UDS,)^18^ which is a sample comprising all HRSA-funded FQHCs that includes annual data on patient characteristics, organizational and structural characteristics, quality of care measures, and service volume. We excluded 31 FQHCs that were located in US territories, 230 FQHCs that were newly established after 2011, and 44 FQHCs where every delivery site was either school based, mobile, or seasonal (eFigure 1 in the Supplement). We further excluded 110 FQHCs that were located in states that expanded Medicaid in 2015 (Indiana, Pennsylvania, and Arkansas) or 2016 (Louisiana and Montana), as our goal was to capture the 5-year effects of expansion.

Boston University’s institutional review board deemed the study exempt and waived informed consent because data were publicly available. This study followed the Strengthening the Reporting of Observational Studies in Epidemiology (STROBE) reporting guidelines.

### Outcomes

We examined 2 sets of outcomes. First, for each FQHC, we measured the percentage of all patients 18 years and older who were uninsured. Second, we measured 2 clinical quality measures that may be sensitive to gaining health insurance coverage under Medicaid expansion: (1) percentage of patients with diabetes age 18 to 75 years with a hemoglobin A_1c_ (HbA_1c_) level of less than or equal to 9% (to convert to the proportion of total hemoglobin, multiply by 0.01) during the most recent visit and (2) percentage of patients with hypertension age 18 to 85 years whose blood pressure was less than 140/90 mm Hg during the most recent visit. The clinical quality measures were assessed for the full population and separately for each racial and ethnic group, in which race and ethnicity was derived from self-reported data in electronic health records (EHRs), with race and ethnicity options defined by the FQHC. All quality measures were reported as numerator and denominator counts in the UDS, as extracted from EHRs, and further defined in the UDS manual (eTable 2 in the Supplement).^19^ Notably, HbA_1c_ levels of 9% or less captured patients who did not have *poor control* based on the definition used in the UDS, herein referred to as *control*, although control is conventionally defined as an HbA_1c_ level of less than 8%.^20^

### Exposure and Covariates

The primary exposure was location in a state that expanded Medicaid by the end of 2014, whereas *nonexposure* was defined as location in a state that had not expanded Medicaid by 2018. eTable 1 in the Supplement lists expansion and nonexpansion states. Covariates included the following time-variant confounding variables: patient-centered medical home (PCMH) accreditation, EHR use, types and levels of grant revenue, and percentage of patients experiencing homelessness and who spoke a primary language other than English.

### Statistical Analyses

The unit of analysis was the FQHC year. First, we descriptively compared baseline FQHC characteristics in expansion vs nonexpansion states. Second, we used a difference-in-differences approach to compare FQHCs located in Medicaid expansion vs nonexpansion states before (2012-2013) vs after (2014-2018) expansion. The difference-in-differences approach allowed for baseline differences between groups, in which FQHCs in expansion states served as their own controls and the comparison group of FQHCs accounted for anticipated secular trends in absence of expansion.

To estimate the 5-year association of Medicaid expansion with uninsurance and quality of care in FQHCs, we used linear probability models to estimate absolute changes in outcomes, which were measured in percentage points (PPs; 0%-100%). In the first specification, all models included an indicator for whether the FQHC was located in an expansion state, an indicator for whether the year was in the preexpansion vs postexpansion period, an interaction term between expansion status and postperiod status (the parameter of interest, or the difference in difference), a vector of time-variant confounding variables as described previously, year fixed effects, state fixed effects, and FQHC-specific fixed effects to account for all time invariant FQHC characteristic that were measured and unmeasured. We used robust variance estimators to account for repeated measures. All models applied analytic weights based on the number of patients in each FQHC’s denominator.

The second model specification included indicators for the number of implementation years following expansion, which was equal to 1 in 2014 and 5 in 2018, rather than an indicator for the entire postperiod. All *P* values were 2-tailed, with a priori statistical significance α = .05. Analyses were performed using Stata, version 15.0 (StataCorp).

### Sensitivity Analyses and Tests of Assumptions

Five states (Connecticut, California, Minnesota, New Jersey, and Washington) and Washington, DC partially expanded Medicaid eligibility under ACA before 2014.^21^ As this may result in conservative effect estimates, we reran all analyses to exclude these states. We also visually and statistically assessed (1) preperiod parallel trends and (2) temporal changes in patient characteristics between the exposure and comparison groups. Finally, as a falsification test, we reran our analyses using 2011 to 2012 as the preperiod and 2013 as the placebo postperiod.

## Results

### Baseline Study Sample Characteristics

Our final study sample included 946 FQHCs per year, representing a mean of 18.9 million patients per year before Medicaid expansion (2012-2013). Of these patients, 64% were age 18 to 64 years, 57% were women, 19% were non-Hispanic Black, 27% were Hispanic, 69% had an income level less than 100% of the FPL, 38% did not have insurance, 34% were enrolled in Medicaid, 9% were experiencing homelessness, and 17% spoke a primary language other than English (Table 1). A total of 416 FQHCs (44%) were located in rural areas, 454 (48%) had PCMH recognition, and 899 (95%) had EHRs at all sites. A total of 578 FQHCs (61.1%) were in Medicaid expansion states, representing 13.0 million patients per year at baseline, and 368 FQHCs (38.9%) were in nonexpansion states, representing 6.0 million patients per year at baseline. In expansion states, patients were less likely to be Black and more likely to be Hispanic, substantially more likely to have Medicaid coverage and less likely to be uninsured, and more likely to be experiencing homelessness and speak a primary language other than English. The FQHCs in expansion states were also less likely to be located in rural areas and more likely to be certified PCMHs.

**Table 1.** Baseline Characteristics of FQHCs by Medicaid Expansion Status From 2012 to 2013

Characteristic	FQHCs, %			Standardized difference
	All (n = 946 FQHCs, with 18 934 190 patients/y)^a^	Expansion state (n = 578 FQHCs, with 12 950 090 patients/y)^a^	Nonexpansion state (n = 368 FQHCs, with 5 984 416 patients/y)^a^	
Patients per FQHC, mean (SD), No.	20 015 (21 341)	22 405 (24 142)	16 262 (15 257)	−0.30
Age, y				
>18	27.2	27.8	26.4	−0.11
18-64	64.4	64.3	64.7	0.04
≥65	8.3	8.0	8.9	0.16
Sex				
Female	57.4	57.0	58.0	0.14
Male	42.6	43.0	42.0	0.14
Race and ethnicity				
Hispanic	27.3	29.9	23.3	−0.24
Non-Hispanic				
American Indian/Alaskan Native	1.4	1.5	1.4	−0.01
Asian	2.6	3.7	0.9	0.15
Black	18.9	15.7	23.9	0.34
White	44.0	42.3	46.8	0.15
Other^b^	4.8	5.8	3.3	−0.29
Income				
Less than 100% federal poverty level	69.0	68.9	69.2	0.02
Less than 200% federal poverty level	92.0	92.1	91.9	−0.03
Insurance coverage among adults				
Uninsured	45.6	40.9	52.9	0.59
Medicaid	22.7	27.5	15.1	−0.98
Medicare	13.0	12.6	13.8	0.15
Private coverage	17.3	17.0	17.7	0.05
Other public coverage	1.9	2.4	0.9	−0.31
Other patient characteristics				
Experiencing homelessness	9.2	.8	6.6	−0.19
Primary language other than English	17.3	19.3	14.1	−0.26
Other health center characteristics				
Rural service area	43.8	37.0	54.4	−0.35
PCMH recognition	48.4	51.6	43.2	−0.17
Electronic health record adoption	94.8	94.7	94.8	0.01
Total grant revenue per patient, mean (SD), $^c^	341 (288)	360 (301)	317 (263)	−0.14

Abbreviations: FQHC, federally qualified health center; PCMH, patient-centered medical home.

^a^ Sample size based on the preperiod (2012-2013). Excludes FQHCs in states that expanded Medicaid in 2015 (Indiana and Pennsylvania) and 2016 (Arkansas, Louisiana, and Montana).

^b^ Other includes Native Hawaiian, Other Pacifier Islander, and patients reporting more than 1 race and ethnicity.

^c^ Includes all sources of federal, state, and local grant revenue. Reported as an annual mean.

### Changes in Insurance Coverage

In states that expanded Medicaid, the percentage of adults who utilized FQHCs who were uninsured declined after expansion, with the largest declines occurring during the initial expansion year, smaller declines during the second expansion year, and no further statistical change in uninsurance rates in years 3 to 5 of expansion (Figure). In expansion states, the uninsurance rate declined from 42.2% (95% CI, 40.5%-43.8%) in 2012 to 21.3% (95% CI, 20.1%-22.5%) by 2018; the concurrent change in nonexpansion states was 53.4% (95% CI, 51.3%-55.5%) in 2012 to 41.9% (95% CI, 39.8%-43.9%) by 2018. Relative to nonexpansion states, there was an 8.77-PP (95% CI, 7.61-9.93) absolute decline in uninsurance by year 1 of expansion and a 9.98-PP (95% CI, 8.58-11.38) absolute decline by year 2 compared with the 2012 to 2013 preperiod. Changes in uninsurance remained stable thereafter, where the pooled 5-year comparative reduction in uninsurance was 9.24 PP (95% CI, 7.94-10.54) (eTable 5 in the Supplement).

**Figure.**
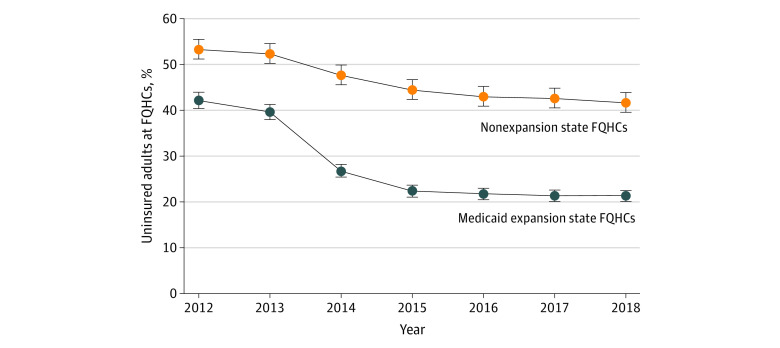
Percentage of Uninsured Adults at Federally Qualified Health Centers (FQHCs) by State Medicaid Expansion Status From 2012 to 2018 The study sample includes 946 unique FQHCs serving a mean of 18 934 190 patients per year in the preperiod (2012-2013) and 21 897 062 patients per year in the postperiod (2014-2018). The sample includes FQHCs in all states except those that expanded Medicaid in 2015 (Indiana and Pennsylvania) and 2016 (Arkansas, Louisiana, and Montana).

### Changes in Hypertension Control

Medicaid expansion was associated with a 1.61-PP increase (95% CI, 0.58-2.64) in the percentage of FQHC patients with hypertension with controlled blood pressure compared with FQHC patients in nonexpansion states across the pooled 5-year expansion period (Table 2). The association of expansion with blood pressure control increased over time; by year 5, expansion was associated with a 2.36-PP (95% CI, 1.01-3.71) comparative increase in patients with controlled blood pressure. The association of expansion with blood pressure control was largest among Black patients (difference in difference by year 5: 3.38 PP; 95% CI, 0.80-5.96) and Hispanic patients (difference in difference by year 5: 3.03 PP; 95% CI, 1.00-5.07). The magnitude of association was also large among American Indian/Alaskan Native patients, although our findings were only statistically significant in year 3 (difference in difference: 7.46 PP; 95% CI, 0.01-14.92).

**Table 2.** Association Between Medicaid Expansion and Hypertension Control in FQHC Patients in Difference-in-Differences Results by Race and Ethnicity^a^

Characteristic	Expansion states^b^		Nonexpansion states^c^		Difference in difference (95% CI)^d^	*P* value
	Preperiod^e^	Postperiod^e^	Preperiod^e^	Postperiod^e^		
All races, %	64.3	64.5	62.1	60.5	NA	NA
Years 1-5					1.61 (0.58 to 2.64)	.002
1	NA	NA	NA	NA	1.17 (0.08 to 2.27)	.04
2					1.08 (−0.16 to 2.32)	.09
3					1.57 (0.26 to 2.87)	.02
4					1.91 (0.58 to 3.23)	.01
5					2.36 (1.01 to 3.71)	.001
White, non-Hispanic, %	65.6	66.4	64.5	63.4	NA	NA
Years 1-5					1.90 (0.58 to 3.22)	.01
1	NA	NA	NA	NA	1.60 (0.22 to 2.97)	.02
2					1.49 (−0.21 to 3.19)	.09
3					2.35 (0.60 to 4.10)	.01
4					2.04 (0.39 to 3.70)	.02
5					2.05 (0.35 to 3.76)	.02
Black, non-Hispanic, %	58.5	58.3	57.2	54.1	NA	NA
Years 1-5					2.24 (0.30 to 4.18)	.02
1	NA	NA	NA	NA	1.14 (−0.96 to 3.24)	.29
2					1.47 (−0.91 to 3.86)	.23
3					2.51 (0.08 to 4.94)	.04
4					2.75 (0.17 to 5.34)	.04
5					3.38 (0.80 to 5.96)	.01
Hispanic, %	65.7	65.8	64.7	63.1	NA	NA
Years 1-5					1.57 (−0.06 to 3.20)	.06
1	NA	NA	NA	NA	0.42 (−1.60 to 2.43)	.69
2					0.86 (−1.11 to 2.84)	.39
3					1.07 (−0.93 to 3.06)	.30
4					2.55 (0.55 to 4.54)	.01
5					3.03 (1.00 to 5.07)	.004
Asian, non-Hispanic, %	69.7	68.5	64.6	64.4	NA	NA
Years 1-5					−0.82 (−4.64 to 3.01)	.68
1	NA	NA	NA	NA	−1.46 (−6.19 to 3.27)	.55
2					−0.78 (−5.18 to 3.61)	.73
3					−1.33 (−5.85 to 3.18)	.56
4					−0.87 (−5.14 to 3.39)	.69
5					0.38 (−3.73 to 4.49)	.86
American Indian/Alaskan Native, non-Hispanic, %	60.7	63.0	61.2	58.1	NA	NA
Years 1-5					6.47 (−0.31 to 13.25)	.06
1	NA	NA	NA	NA	7.32 (−1.10 to 15.74)	.09
2					5.46 (−1.23 to 12.16)	.11
3					7.46 (0.01 to 14.92)	.05
4					4.88 (−2.23 to 11.99)	.18
5					7.08 (−0.51 to 14.67)	.07

Abbreviations: FQHC is federally qualified health center; NA, not applicable.

^a^ Hypertension control was defined as the percentage of adult patients with hypertension whose blood pressure was less than 140/90 mm Hg during the most recent visit.

^b^ A total of 578 unique Medicaid expansion state FQHCs serving 12 950 090 patients per year in the preperiod and 14 910 871 patients per year in the postperiod. This included a mean of 2 266 960 patients with hypertension per year (all races and ethnicities), including 928 008 non-Hispanic White patients per year, 406 113 non-Hispanic Black patients per year, 686 825 Hispanic patients per year, 115 481 Asian patients per year, and 14 843 American Indian/Alaskan Native patients per year with hypertension.

^c^ A total of 368 unique nonexpansion state FQHCs serving 5 984 416 patients per year in the preperiod and 6 718 066 patients per year in the postperiod. This included a mean of 1 282 656 patients with hypertension per year (all races and ethnicities), including 537 062 non-Hispanic White patients per year, 445 014 non-Hispanic Black patients per year, 244 086 Hispanic patients per year, 11 743 Asian patients per year, and 9760 American Indian/Alaskan Native patients per year with hypertension.

^d^ Difference-in-difference results for years 1 to 5 represent the adjusted, absolute percentage point difference (0-100 scale) in the percentage of patients with hypertension control between Medicaid expansion vs nonexpansion states in the preperiod (2012-2013) vs postperiod (2014-2018). Difference-in-difference results in individual years *x* represent the effect after *x* years of postimplementation time.

^e^ Preperiod and postperiod rates represent unadjusted estimates.

### Changes in Diabetes Control

Medicaid expansion was associated with a 1.84-PP increase (95% CI, 0.71-2.98) in the percentage of FQHC patients with diabetes with HbA_1c_ levels of 9% or less compared with FQHC patients in nonexpansion states across the pooled 5-year expansion period (Table 3). The magnitude of the association peaked in year 3 (difference in difference: 3.14 PP; 95% CI, 1.49-4.80). When examining changes across racial and ethnic groups, expansion was associated with comparative improvements in glucose control among Black (difference in difference in year 5: 3.88 PP; 95% CI, 0.86-6.90) and Hispanic (difference in difference in year 5: 2.93 PP; 95% CI, 0.58-5.27) patients, for whom a statistically significant result was not observable until year 2 and 3, respectively. Expansion was also associated with statistically significant improvements in glucose control among White patients in years 3 to 4; however, no statistically significant associations were observed among Asian or American Indian/Alaskan Native patients.

**Table 3.** Association Between Medicaid Expansion and Diabetes Control in FQHCs Patients in Difference-in-Differences Results by Race and Ethnicity^a^

Characteristic	Expansion states^b^		Non-expansion states^c^		Difference in difference (95% CI)^d^	*P* value
	Preperiod^e^	Postperiod^e^	Preperiod^e^	Postperiod^e^		
All races, %	69.5	69.1	69.6	67.4	NA	NA
Years 1-5					1.84 (0.71 to 2.98)	.002
1	NA	NA	NA	NA	0.85 (−0.68 to 2.38)	.28
2					1.16 (−0.38 to 2.70)	.14
3					3.14 (1.49 to 4.80)	<.001
4					2.43 (0.98 to 3.88)	.001
5					1.68 (0.18 to 3.19)	.03
White, non-Hispanic, %	72.7	71.7	72.5	70.1	NA	NA
Years 1-5					1.43 (0.06 to 2.80)	.04
1	NA	NA	NA	NA	−0.08 (−2.07 to 1.90)	.94
2					0.01 (−1.80 to 1.81)	.99
3					3.16 (1.19 to 5.13)	.002
4					2.82 (1.05 to 4.60)	.002
5					1.36 (−0.50 to 3.21)	.15
Black, non-Hispanic, %	65.2	65.9	68.1	65.1	NA	NA
Years 1-5					3.89 (1.62 to 6.17)	.001
1	NA	NA	NA	NA	2.73 (−0.35 to 5.80)	.08
2					4.27 (1.48 to 7.06)	.003
3					4.93 (1.75 to 8.11)	.002
4					3.70 (0.86 to 6.54)	.01
5					3.88 (0.86 to 6.90)	.01
Hispanic, %	66.1	66.0	65.6	63.9	NA	NA
Years 1-5					2.02 (0.10 to 3.94)	.04
1	NA	NA	NA	NA	0.50 (−2.08 to 3.08)	.70
2					0.94 (−1.68 to 3.57)	.48
3					3.12 (0.57 to 5.67)	.02
4					2.70 (0.43 to 4.97)	.02
5					2.93 (0.58 to 5.27)	.02
Asian, non-Hispanic, %	77.9	79.5	73.6	73.0	NA	NA
Years 1-5					1.93 (−3.85 to 7.71)	.51
1	NA	NA	NA	NA	3.47 (−2.89 to 9.82)	.29
2					−1.92 (−8.26 to 4.43)	.55
3					2.04 (−4.28 to 8.36)	.53
4					2.35 (−3.90 to 8.60)	.46
5					3.49 (−3.29 to 10.27)	.31
American Indian/Alaskan Native, non-Hispanic, %	61.1	62.7	61.9	60.4	NA	NA
Years 1-5					4.82 (−2.30 to 11.93)	.18
1	NA	NA	NA	NA	7.17 (−2.01 to 16.35)	.13
2					6.81 (−11.38 to 25.00)	.46
3					1.93 (−7.23 to 11.09)	.68
4					3.51 (−3.72 to 10.75)	.34
5					4.59 (−3.21 to 12.40)	.25

Abbreviations: FQHC, federally qualified health center; NA, not applicable.

^a^ Diabetes control was defined as the percentage of adult patients with diabetes with a hemoglobin A_1c_ level of less than or equal to 9% (to convert to the proportion of total hemoglobin, multiply by 0.01) during the most recent visit.

^b^ A total of 578 unique Medicaid expansion state FQHCs serving 12 950 090 patients per year in the preperiod and 14 910 871 patients per year in the postperiod. This included a mean of 1 153 003 patients with diabetes per year (all races), including 362 527 non-Hispanic White patients per year, 172 185 non-Hispanic Black patients per year, 482 419 Hispanic patients per year, 60 250 Asian patients per year, and 3179 American Indian/Alaskan Native patients per year with diabetes

^c^ A total of 368 unique nonexpansion state FQHCs serving 5 984 416 patients per year in the preperiod and 6 718 066 patients per year in the postperiod. This included a mean of 621 372 patients with diabetes per year (all races), including 224 747 non-Hispanic White patients per year, 193 206 non-Hispanic Black patients per year, 171 942 Hispanic patients per year, 7311 Asian patients per year, and 1042 American Indian/Alaskan Native patients per year with diabetes.

^d^ Difference-in-difference results for years 1-5 represent the adjusted, absolute percentage point difference (0-100 scale) in the percentage of patients with hypertension control between Medicaid expansion vs nonexpansion states in the pre- (2012-2013) vs postperiod (2014-2018). Difference-in-difference results in individual years *x* represent the effect after *x* years of postimplementation time. For example, year 5 represents the difference-in-difference in 2018 vs the preperiod.

^e^ Pre/post rates represent unadjusted estimates.

### Sensitivity Analyses

Sensitivity analyses were largely consistent with the main results (eTables 6-9 and eFigures 2-12 in the Supplement). However, changes for some outcomes were nonsignificant after excluding FQHCs in early expansion states.

## Discussion

We found that after 5 years of implementation, Medicaid expansion was associated with large, sustained reductions in uninsurance and improvements in blood pressure and glucose control measures in a nationally representative sample of FQHCs. The association between Medicaid expansion and improvement in health outcomes increased over the 5-year postexpansion period, during which time Black and Hispanic patients experienced substantial gains in blood pressure and glucose control.

We observed larger associations between expansion and health outcome measures in the long vs short term following expansion, suggesting that intermediate outcomes, such as blood pressure and glucose control, may take time to improve following coverage expansions. Once patients gain access to health insurance coverage, they are more likely to use primary care services offered by FQHCs,^22^ despite the fact that FQHCs provide care without concern for ability to pay. Further, insurance also improves access to specialty care and prescription drugs.^22,23,24^ However, establishing regular access to care and implementing an effective care management plan take time; this may especially be the case for patients with new diagnoses of uncontrolled blood pressure or glucose levels. Medicaid expansion may also improve the financial revenues of FQHCs,^8,9^ which may allow FQHCs to expand capacity and invest in quality improvement interventions for all patients. It may take multiple years for additional revenue to manifest into expanded capacity.

While evidence on the association of Medicaid expansion with racial and ethnic disparities has been mixed,^25,26,27,28,29,30,31,32^ Black and Hispanic patients within FQHCs may have been more likely to gain insurance in Medicaid expansion states or may have disproportionately benefited from having insurance postexpansion. The data did not allow us to examine these hypotheses, although other research suggests that expansion was associated with reduced disparities in uninsurance at FQHCs.^33^ Black and Hispanic FQHC patients also experienced larger disparities in outcomes at baseline compared with other racial groups; therefore, they may have had more opportunity for improvement following expansion. Nonetheless, while we observed comparative gains in health outcome measures for Hispanic and Black patients, racial and ethnic disparities in blood pressure control and hypertension control among FQHC patients persist, as described in earlier work.^17,34^ This finding underscores that insurance alone will not eliminate disparities in health outcomes, as Black, Hispanic, and American Indian/Alaskan Native patients face systemic inequities and structural racism that make it especially difficult to control blood pressure and glucose levels.^35,36^

While we observed comparative improvements in health outcomes that were associated with expansion, these gains reflect relatively stable outcomes in expansion states vs worsening trends in outcomes in nonexpansion states. Previous work on FQHCs similarly demonstrated that quality differences in hypertension control and cervical cancer screening were explained by continued decreases in quality measures at FQHCs in nonexpansion states.^4^ This pattern suggests that while intermediate clinical outcomes were slightly worsening in FQHCs in expansion and nonexpansion states before expansion (eFigures 8-12 in the Supplement), expansion may have stabilized these outcomes or slowed the rate of decline. Stable health insurance coverage because of Medicaid expansion may reduce discontinuities in care while also enabling better access to comprehensive care for new patients. These are both especially important for low-income patients with chronic conditions.^37^

Medicaid expansion has been associated with reductions in self-reported rates of psychological distress, whereas evidence on self-reported health status has been mixed.^38,39,40,41^ Other studies that examined the 3 years following expansion have demonstrated lower mortality among patients with end-stage kidney disease and cancer as well as lower infant mortality and cardiovascular mortality in middle-aged adults.^42,43,44,45^ While there has been limited evidence to date about the association of Medicaid expansion with blood pressure control or glucose control, other work has found that expansion was associated with an improvement in quality of care process measures that may be associated with hypertension and diabetes control, such as improved glucose monitoring rates, identification of diabetes and hypertension cases, and other diabetes management measures^41,46,47,48,49,50,51,52,53^ in addition to improved access to diabetes, hypertension, and cardiovascular-related medications.^53,54,55^ The Oregon Health Insurance Experiment, a randomized clinical trial that examined Medicaid insurance, failed to detect improvements in blood pressure or glucose control (likely because of insufficient statistical power) but found increases in diabetes diagnoses and the use of diabetic medications; this study followed patients for 25 months and was limited to 1 state.^56^ A recent study on 178 FQHCs found that in the 2 years following ACA-related expansions, newly insured patients with diabetes at FQHCs experienced reductions in glucose and blood pressure levels.^10^ Another study using national survey data found that after 3 years, expansion was associated with lower systolic blood pressure and blood glucose levels in low-income adults, with no change in diastolic blood pressure or cholesterol levels.^57^ We add to the existing evidence base by examining the longer-term associations of Medicaid with key intermediate health outcomes and by focusing on health care networks that were especially affected by expansion: FQHCs.

Our findings have policy and research implications. First, this study suggests that expanding Medicaid eligibility in nonexpansion states may improve key health outcome measures for many patients with chronic conditions. For example, a 2-PP increase in hypertension control across all FQHCs equates to nearly 100 000 additional patients with hypertension at FQHCs with blood pressure control. This is particularly important as nonexpansion states consider new incentives to expand Medicaid under the 2021 American Rescue Plan.^58^ Second, Medicaid expansion may improve health outcomes in racial and ethnic groups that experience greater levels of health disparities. However, given persistent racial and ethnic disparities in health outcome measures after expansion, access to coverage must be accompanied by targeted investments in the quality of care delivered to racial and ethnic minority populations at FQHCs and the social determinants of health that go beyond what FQHCs may reasonably address. Lastly, continued monitoring and evaluation of Medicaid expansion will be critical, particularly for clinical outcomes that may take time to be affected by insurance coverage.

### Limitations

Our study has limitations. First, UDS data were reported at the FQHC level, so we were unable to adjust for individual-level factors and could not account for patient movement in and out of FQHCs. This likely resulted in conservative estimates if some newly insured patients leave the FQHC, although earlier work suggests that patients continue to use safety net facilities after gaining coverage.^59^ We also did not observe differential changes in patient characteristics over time besides insurance status (eTables 3-4 and eFigure 2 in the Supplement). A lack of patient-level data meant that we were unable to separate how gains in insurance coverage for patients vs gains in patient revenue for FQHCs were associated with the study outcomes. Future studies that use patient-level data are needed to better quantify these mechanisms. Second, the study’s preintervention period was limited to 2 years; we use 2012 as the first year of data because of reporting changes in UDS between 2011 and 2012. Third, UDS data only capture patients who are in some way engaged in care at the FQHC, which may attenuate observed changes in quality measures if patients who were previously unengaged or without a diagnosis newly seek care following expansion. Fourth, we were unable to separately report changes in insurance coverage by race and ethnicity, as these data are not stratified in the UDS. Fifth, sample sizes were limited for Asian and American Indian/Alaskan Native patients; we included these results given underrepresentation of these populations in research. Sixth, data are subject to measurement error, but error is unlikely to change differentially between expansion vs nonexpansion FQHCs. Finally, residual confounding may exist, particularly if there were policy or delivery system changes that occurred during the postperiod that were not associated with expansion and differentially affected expansion vs nonexpansion FQHCs.

## Conclusions

In this nationally representative study of patients in FQHCs, the first 5 years of Medicaid expansion were associated with large, sustained reductions in uninsurance and relative improvements in blood pressure and glucose control measures, including for Hispanic and Black patients. These findings suggest that over the long term, expanding Medicaid in all states may improve chronic disease health outcome measures at FQHCs, which provide care to millions of underserved patients.

## Notes

Supplement. **eFigure 1**. Study sample selection criteria **eTable 1.** Sample sizes by treatment status **eTable 2.** Study measure definitions **eTable 3. **Characteristics of FQHCs by Medicaid expansion status: pre vs. post-period **eTable 4. **Changes in characteristics of FQHCs in Medicaid expansion vs. non-expansion state FQHCs before vs. after expansion: difference-in-difference results (2012-2018) **eFigure 2. **Percent of patients by Medicaid expansion status (2012-2018) **eTable 5. **Association between Medicaid expansion and insurance coverage in FQHC adults: difference-in-differences results **eFigure 3. **Association between Medicaid expansion and insurance coverage in FQHC adults: difference-in-differences results **eTable 6. **Association between Medicaid expansion and hypertension control in FQHC patients: difference-in-differences results—excluding early expansion states **eTable 7. **Association between Medicaid expansion and diabetes control in FQHC patients: difference-in-differences results—excluding early expansion states **eTable 8. **Association between Medicaid expansion and intermediate quality outcomes in FQHC patients: difference-in-differences results with placebo post period **eTable 9. **Interaction between year and expansion status: 2013 vs. 2012 **eFigure 4. **Percent patients with hypertension control in pre-period (all) **eFigure 5. **Percent patients with diabetes control in pre-period (all) **eFigure 6. **Number of visits for diabetes per FQHC over a longer pre-period (2009-2018) **eFigure 7. **Number of visits for hypertension per FQHC over a longer pre-period (2009-2018) **eFigure 8. **Percent FQHC patients with hypertension control by Medicaid expansion status (adjusted)—all races **eFigure 9. **Percent FQHC patients with hypertension control by Medicaid expansion status (adjusted)—Black, non-Hispanic **eFigure 10. **Percent FQHC patients with hypertension control by Medicaid expansion status (adjusted)—Hispanic **eFigure 11. **Percent FQHC patients with diabetes control by Medicaid expansion status (adjusted)—all races **eFigure 12. **Percent FQHC patients with diabetes control by Medicaid expansion status (adjusted)—Black, non-Hispanic
