# Supplementary material for: Association of Medicaid Expansion With 5-Year Changes in Hypertension and Diabetes Outcomes at Federally Qualified Health Centers
Source: JAMA Health Forum. 2021 Sep 10;2(9):e212375. doi: 10.1001/jamahealthforum.2021.2375 (PMC8796924; doi:10.1001/jamahealthforum.2021.2375)
Supplement: Supplement. — eFigure 1. Study sample selection criteria eTable 1. Sample sizes by treatment status eTable 2. Study measure definitions eTable 3. Characteristics of FQHCs by Medicaid expansion status: pre vs. post-period eTable 4. Changes in characteristics of FQHCs in Medicaid expansion vs. non-expansion state FQHCs before vs. after expansion: difference-in-difference results (2012-2018) eFigure 2. Percent of patients by Medicaid expansion status (2012-2018) eTable 5. Association between Medicaid expansion and insurance coverage in FQHC adults: difference-in-differences results eFigure 3. Association between Medicaid expansion and insurance coverage in FQHC adults: difference-in-differences results eTable 6. Association between Medicaid expansion and hypertension control in FQHC patients: difference-in-differences results—excluding early expansion states eTable 7. Association between Medicaid expansion and diabetes control in FQHC patients: difference-in-differences results—excluding early expansion states eTable 8. Association between Medicaid expansion and intermediate quality outcomes in FQHC patients: difference-in-differences results with placebo post period eTable 9. Interaction between year and expansion status: 2013 vs. 2012 eFigure 4. Percent patients with hypertension control in pre-period (all) eFigure 5. Percent patients with diabetes control in pre-period (all) eFigure 6. Number of visits for diabetes per FQHC over a longer pre-period (2009-2018) eFigure 7. Number of visits for hypertension per FQHC over a longer pre-period (2009-2018) eFigure 8. Percent FQHC patients with hypertension control by Medicaid expansion status (adjusted)—all races eFigure 9. Percent FQHC patients with hypertension control by Medicaid expansion status (adjusted)—Black, non-Hispanic eFigure 10. Percent FQHC patients with hypertension control by Medicaid expansion status (adjusted)—Hispanic eFigure 11. Percent FQHC patients with diabetes control by Medicaid expansion status (adjusted)—all r [file jamahealthforum-e212375-s001.pdf]

Supplemental Online Content

Cole MB, Kim J-H, Levengood TW, Trivedi AN. Association of Medicaid expansion with 5-year changes in hypertension and diabetes outcomes at federally qualified health centers. *JAMA Health Forum*. 2021;2(9):e212375. doi:10.1001/jamahealthforum.2021.2375

eFigure 1. Study sample selection criteria

eTable 1. Sample sizes by treatment status

eTable 2. Study measure definitions

eTable 3. Characteristics of FQHCs by Medicaid expansion status: pre vs. post-period

eTable 4. Changes in characteristics of FQHCs in Medicaid expansion vs. non-expansion state FQHCs before vs. after expansion: difference-in-difference results (2012-2018)

eFigure 2. Percent of patients by Medicaid expansion status (2012-2018)

eTable 5. Association between Medicaid expansion and insurance coverage in FQHC adults: difference-in-differences results

eFigure 3. Association between Medicaid expansion and insurance coverage in FQHC adults: difference-in-differences results

eTable 6. Association between Medicaid expansion and hypertension control in FQHC patients: difference-in-differences results—excluding early expansion states

eTable 7. Association between Medicaid expansion and diabetes control in FQHC patients: difference-in-differences results—excluding early expansion states

eTable 8. Association between Medicaid expansion and intermediate quality outcomes in FQHC patients: difference-in-differences results with placebo post period

eTable 9. Interaction between year and expansion status: 2013 vs. 2012

eFigure 4. Percent patients with hypertension control in pre-period (all)

eFigure 5. Percent patients with diabetes control in pre-period (all)

eFigure 6. Number of visits for diabetes per FQHC over a longer pre-period (2009-2018)

eFigure 7. Number of visits for hypertension per FQHC over a longer pre-period (2009-2018)

eFigure 8. Percent FQHC patients with hypertension control by Medicaid expansion status (adjusted)—all races

eFigure 9. Percent FQHC patients with hypertension control by Medicaid expansion status (adjusted)—Black, non-Hispanic

eFigure 10. Percent FQHC patients with hypertension control by Medicaid expansion status (adjusted)—Hispanic

eFigure 11. Percent FQHC patients with diabetes control by Medicaid expansion status (adjusted)—all races

eFigure 12. Percent FQHC patients with diabetes control by Medicaid expansion status (adjusted)—Black, non-Hispanic

This supplemental material has been provided by the authors to give readers additional information about their work.

© 2021 Cole MB et al. *JAMA Health Forum.*

A. Study sample selection

eFigure 1. Study sample selection criteria

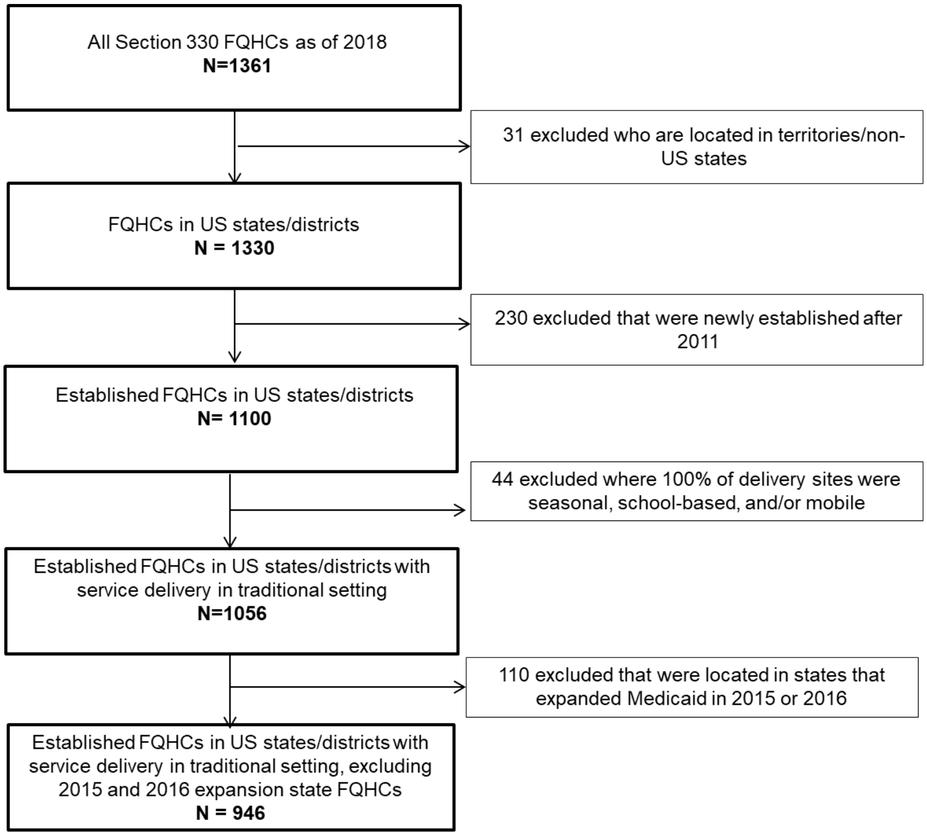

© 2021 Cole MB et al. *JAMA Health Forum.*

B. Medicaid expansion definition and sample sizes

Our treatment group included all 578 FQHCs in states that had expanded Medicaid expansion by the end of 2014, inclusive of Michigan and New Hampshire, which expanded in April 2014 and August 2014, respectively. This group also included FQHCs in five states (California, Connecticut, Minnesota, New Jersey, and Washington) and the District of Columbia that expanded or partially expanded Medicaid eligibility prior to 2014; we included these states in our main analyses because the largest changes in enrollment for these states occurred in 2014. Our control group included 368 FQHCs in states that had not expanded Medicaid eligibility as of the end of 2014. Pennsylvania (2/2015), Indiana (1/2015), Alaska (9/2015), Montana (1/1/2016), and Louisiana (7/1/2016) expanded during our study period, but after 2014, and thus were excluded. We excluded these states because our primary interest was the five-year effect of Medicaid expansion, rather than the average effect over a time-weighted post-period.

eTable 1. Sample Sizes by Treatment Status

Medicaid Expansion States	# FQHCs in sample	# patients in 2018	Non-Medicaid Expansion States	# FQHCs in sample	# patients in 2018
AR	12	222,844	AL	12	306,002
AZ	16	605,891	FL	39	1,599,794
CA	117	4,360,522	GA	23	489,080
CO	14	605,944	ID	11	169,648
CT	13	363,319	KS	13	206,302
DC	4	171,483	ME	17	184,501
DE	3	48,652	MO	20	482,400
HI	14	157,097	MS	21	322,686
IA	13	211,924	NC	27	491,355
IL	37	1,281,941	NE	6	97,773
KY	19	472,913	OK	17	232,518
MA	35	757,065	SC	18	387,641
MD	14	324,403	SD	6	101,792
MI	28	619,935	TN	23	377,723
MN	15	168,604	TX	60	1,366,506
NH	10	40,272	UT	11	158,909
ND	4	92,624	VA	24	328,144
NJ	19	528,990	WI	15	284,578
NM	15	332,709	WY	5	22,651
NV	2	73,792			
NY	49	2,034,294			
OH	32	622,589			
OR	25	359,580			
RI	8	178,033			
VT	8	160,602			
WA	25	1,184,526			
WV	27	470,451			
Total	578	16,453,649	Total	368	8,805,994

Note: sample sizes reflect total number of unique patients served by the FQHCs

© 2021 Cole MB et al. *JAMA Health Forum.*

C. Outcome measure definitions

All measures were obtained from the 2012-2018 Uniform Data System (UDS), which was acquired through a Freedom of Information Act (FOIA) request to the Health Resources and Services Administration (HRSA).

eTable 2. Study measure definitions

Uninsured	Percentage of all adult patients who did not have medical insurance	All unique patients age 18+ who had a medical visit at the FQHC during the measurement year.	Patients age 18+ who did not have medical insurance at the time of their last visit.	None.	Classify patients as uninsured if their visit was self-pay or paid for by a third-party source that was not an insurance, such as EPSDT, BCCCP, Title X, or some state or local safety net or indigent care programs. Do not count patients as uninsured if their medical insurance did not pay for their visit.
Controlled hypertension	Percentage of patients 18–85 years of age who had a diagnosis of hypertension and whose blood pressure (BP) was Adequately controlled (less than 140/90 mmHg) during the measurement period	Patients 18 through 85 years of age who had a diagnosis of essential hypertension within the first six months of the measurement period or any time prior to the measurement period with a medical visit during the measurement period	Patients whose blood pressure at the most recent visit is adequately Controlled (systolic blood pressure less than 140 mmHg and diastolic blood pressure less than 90 mmHg) during the measurement period	Denominator<br>◦ Patients with evidence of end stage renal disease (ESRD), dialysis or renal transplant before or during the measurement period<br>◦ Patients with a diagnosis of pregnancy during the measurement period<br>◦ Patients who were in hospice care during the measurement period	◦ Only blood pressure readings performed by a clinician in the provider office are acceptable for numerator compliance with this measure<br>◦ If no blood pressure is recorded during the measurement period, the patient's blood pressure is assumed "not controlled."<br>◦ If there are multiple blood pressure readings on the same day, use the lowest systolic and the lowest diastolic reading as the most recent blood pressure reading.
"Controlled" diabetes	Percentage of patients 18–75 years of age with diabetes who had hemoglobin A1c (HbA1c) less than or equal to 9.0 percent during the measurement period	Patients 18 through 75 years of age with diabetes with a medical visit during the measurement period, including those who had no test conducted during the measurement period	Patients whose most recent HbA1c level performed during the measurement year is <= 9.0 percent	Denominator<br>◦ Patients who were in hospice care during the measurement period	• Include patients in the numerator whose most recent HbA1c level is <= 9 percent; if the most recent HbA1c result is missing or if no HbA1c tests were performed or documented during the measurement period, then the patient is in the denominator but not the numerator.<br>• Only include patients with an active diagnosis of Type 1 or Type 2 diabetes in the denominator.<br>• Do not include patients with a diagnosis of secondary diabetes due to another condition in the denominator.

Notes: For details on each measure and how to calculate each numerator and denominator, please see the HRSA UDS manuals, available at http://www.bphc.hrsa.gov/datareporting/reporting/index.html.The “controlled” diabetes measure is based on the percent of patients who did not have poor control (HbA1C >9%).

© 2021 Cole MB et al. *JAMA Health Forum.*

D. Population characteristics over time

eTable 3. Characteristics of FQHCs by Medicaid Expansion Status: pre vs. post-period

	Pre-Period (2012-2013)				Post-Period (2014-2018)			
	All CHCs[a]	Expansion State CHCs[b]	Non-Expansion State CHCs[c]	standardized difference	All CHCs[a]	Expansion State CHCs[b]	Non-Expansion State CHCs[c]	standardized difference
**Mean patients per center**	20015	22405	16262	-0.3042	23147	26004	18610	-0.309
**Age (%)**								
Under 18	27.2%	27.8%	26.4%	-0.109	27.1%	27.5%	26.3%	-0.097
18-64	64.4%	64.3%	64.7%	0.036	63.2%	63.0%	63.4%	0.030
65 or older	8.3%	8.0%	8.9%	0.157	9.8%	9.5%	10.3%	0.134
**Sex (%)**								
Female	57.4%	57.0%	58.0%	0.143	56.9%	56.4%	57.6%	0.196
**Race/ethnicity (%)**								
White, non-Hispanic	44.0%	42.3%	46.8%	0.145	43.0%	41.4%	45.4%	0.135
Black, non-Hispanic	18.9%	15.7%	23.9%	0.344	18.6%	15.5%	23.7%	0.350
Asian, non-Hispanic	2.6%	3.7%	0.9%	-0.361	2.8%	4.0%	1.0%	-0.397
AIAN, non-Hispanic	1.4%	1.5%	1.4%	-0.014	1.4%	1.4%	1.2%	-0.029
Other race, non-Hispanic	4.8%	5.8%	3.3%	-0.289	5.0%	6.0%	3.5%	-0.293
Hispanic	27.3%	29.9%	23.3%	-0.242	28.3%	30.8%	24.4%	-0.240
**Income (%)**								
Under 100% federal poverty	69.0%	68.9%	69.2%	0.018	66.7%	65.9%	68.1%	0.125
Under 200% federal poverty	92.0%	92.1%	91.9%	-0.026	91.2%	91.0%	91.5%	0.043
**Insurance coverage,**								
Uninsured	45.6%	40.9%	52.9%	0.59	30.9%	22.7%	43.9%	1.21
Medicaid	22.7%	27.5%	15.1%	-0.98	32.8%	43.1%	16.5%	-2.02
Medicare	13.0%	12.6%	13.8%	0.15	14.5%	14.1%	15.1%	0.11
Private coverage	17.3%	17.0%	17.7%	0.05	21.3%	19.6%	23.9%	0.34
Other public coverage	1.9%	2.4%	0.9%	-0.31	0.6%	0.6%	0.7%	0.05
**Other patient**								
Experiencing homelessness	9.2%	10.8%	6.6%	-0.192	8.2%	9.4%	6.2%	-0.158
Primary language other	17.3%	19.3%	14.1%	-0.256	17.8%	19.9%	14.5%	-0.276
**Other health center**								
Rural service area	43.8%	37.0%	54.4%	-0.353	43.5%	37.0%	53.8%	-0.343
Patient centered medical	48.4%	51.6%	43.2%	-0.169	81.3%	84.1%	76.8%	-0.185
Electronic Health Record	94.8%	94.7%	94.8%	0.005	99.1%	99.3%	98.8%	-0.054
Total grant revenue/patient	$341	$360	$317	-0.141	$393	$404	$375	-0.100

Abbreviations: FQHC is federally qualified health center. SD is standard deviation. AIAN is American Indian/Alaskan Native. PCMH is patient centered medical home.

[a] N=946 unique CHCs representing 18,934,190 patients/year in the pre-period and 21,897,062 patients/year in the post-period. Sample excludes FQHCs in states that expanded Medicaid in 2015 (IN, PA) and 2016 (AK, LA, MT)

[b] N=578 unique Medicaid expansion state CHCs representing 12,950,090 patients/year in the pre-period and 15,030,213 patients/year in the post-period

[c] N=368 unique non-expansion state CHCs representing 5,984,416 patients/year in the pre-period and 6,848,480 patients/year in the post-period.

© 2021 Cole MB et al. *JAMA Health Forum.*

eTable 4. Changes in Characteristics of FQHCs in Medicaid expansion vs. non-expansion state FQHCs before vs. after expansion: difference-in-difference results (2012-2018)

	Coef.[a]	Robust SE	t	P>|t|	[95%	CI]
Percent age 65+	-0.0888	0.1066	-0.83	0.405	-0.2980	0.1203
Percent White, non-Hispanic	0.4064	0.3217	1.26	0.207	-0.2250	1.0378
Percent Hispanic	-0.3473	0.2481	-1.40	0.162	-0.8342	0.1396
Percent Black, non-Hispanic	0.2470	0.2026	1.22	0.223	-0.1506	0.6445
Percent female	-0.2996	0.1562	-1.92	0.055	-0.6061	0.0069
Percent under 200% FPL	-0.7328	0.5915	-1.24	0.216	-1.8936	0.4279
Percent with primary lang other than English	-0.5673	0.6083	-0.93	0.351	-1.7610	0.6264
Percent experiencing homelessness	-0.2448	0.3166	-0.77	0.440	-0.8660	0.3765
Number of hypertensive patients/FQHC	0.0200	0.0197	1.01	0.310	-0.0187	0.0587
Number of diabetic patients/FQHC	0.0191	0.0195	0.98	0.328	-0.0192	0.0574

[a] All coefficients represent the difference-in-difference between expansion vs. non-expansion state FQHCs in the pre- (2012-2013) vs. the post-period (2014-2018). All models are fixed effects models that adjust for year and cluster errors at the FQHC-level. Outcomes that reflect the percentage of patients are modelled using linear probability models whereas count outcomes (number of patients) are modelled using Poisson models.

© 2021 Cole MB et al. *JAMA Health Forum.*

eFigure 2-a. Percent of patients age 65+ by Medicaid expansion status (2012-2018)

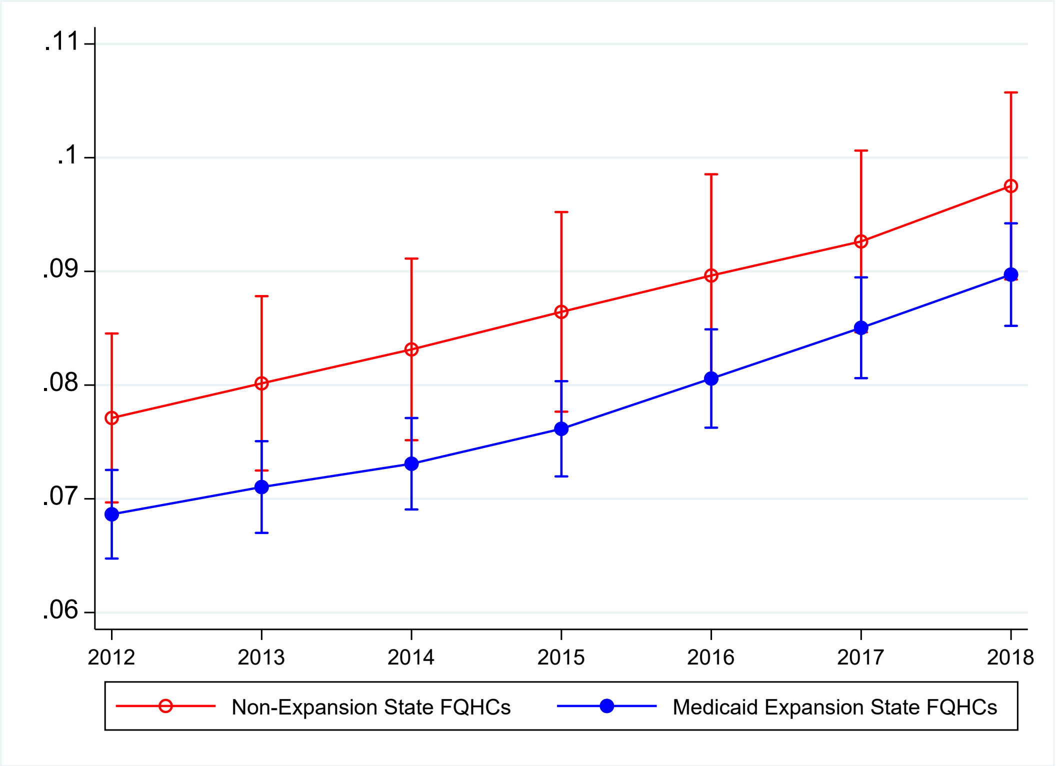

© 2021 Cole MB et al. *JAMA Health Forum.*

eFigure 2-b. Percent of patients who were non-Hispanic White by Medicaid expansion status (2012-2018)

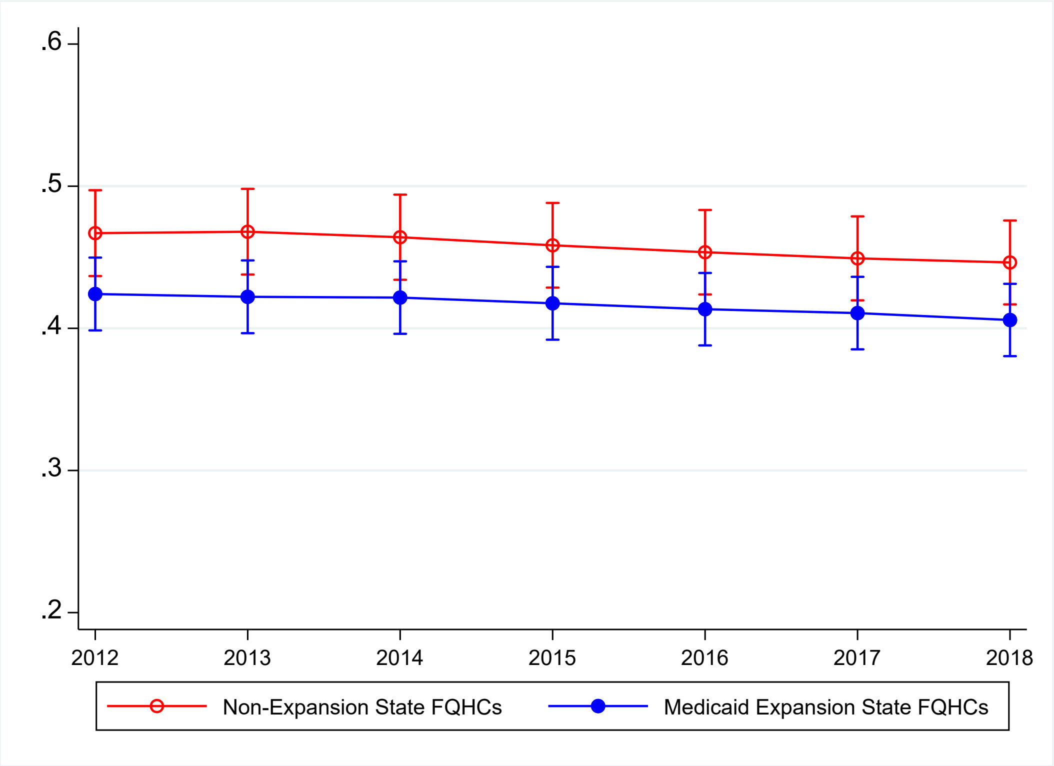

© 2021 Cole MB et al. *JAMA Health Forum.*

eFigure S2-c. Percent of patients who were Hispanic by Medicaid expansion status (2012-2018)

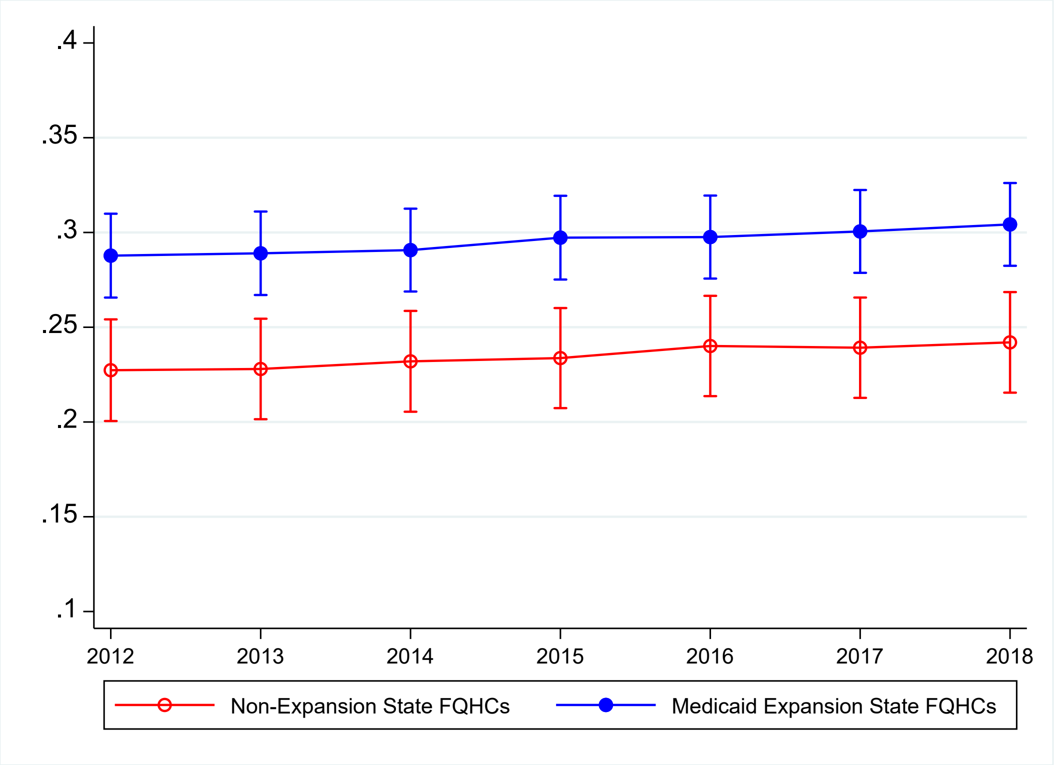

© 2021 Cole MB et al. *JAMA Health Forum.*

eFigure S2-d. Percent of patients who were non-Hispanic Black by Medicaid expansion status (2012-2018)

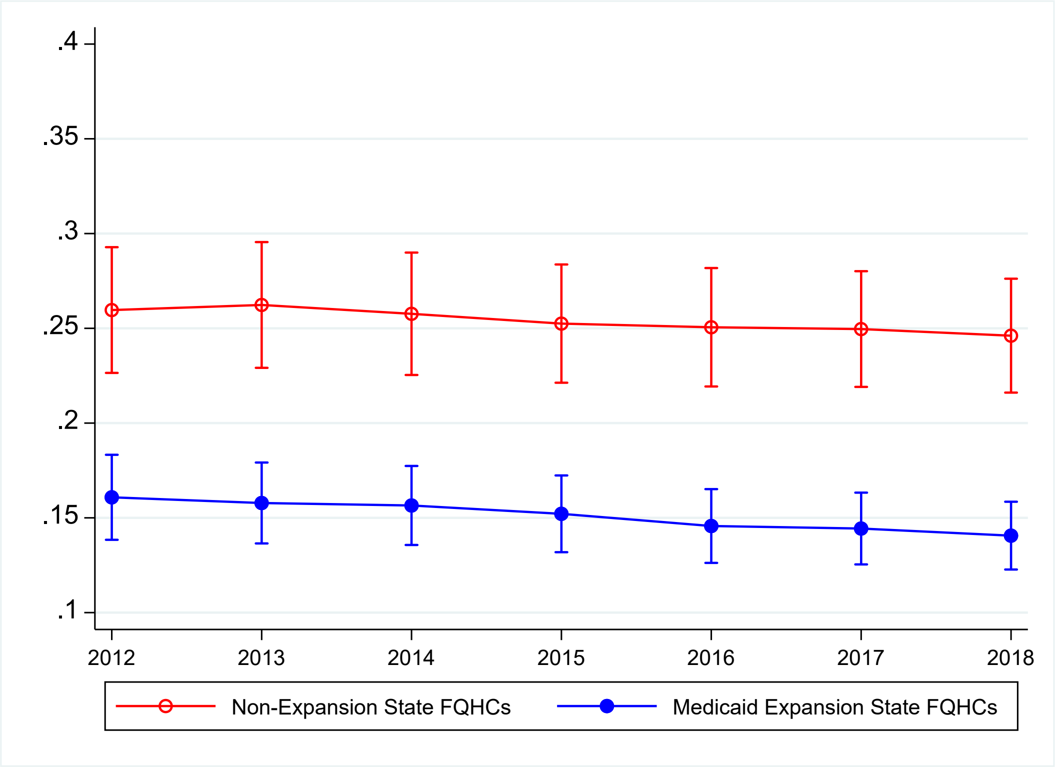

© 2021 Cole MB et al. *JAMA Health Forum.*

eFigure S2-e. Percent of patients who were female by Medicaid expansion status (2012-2018)

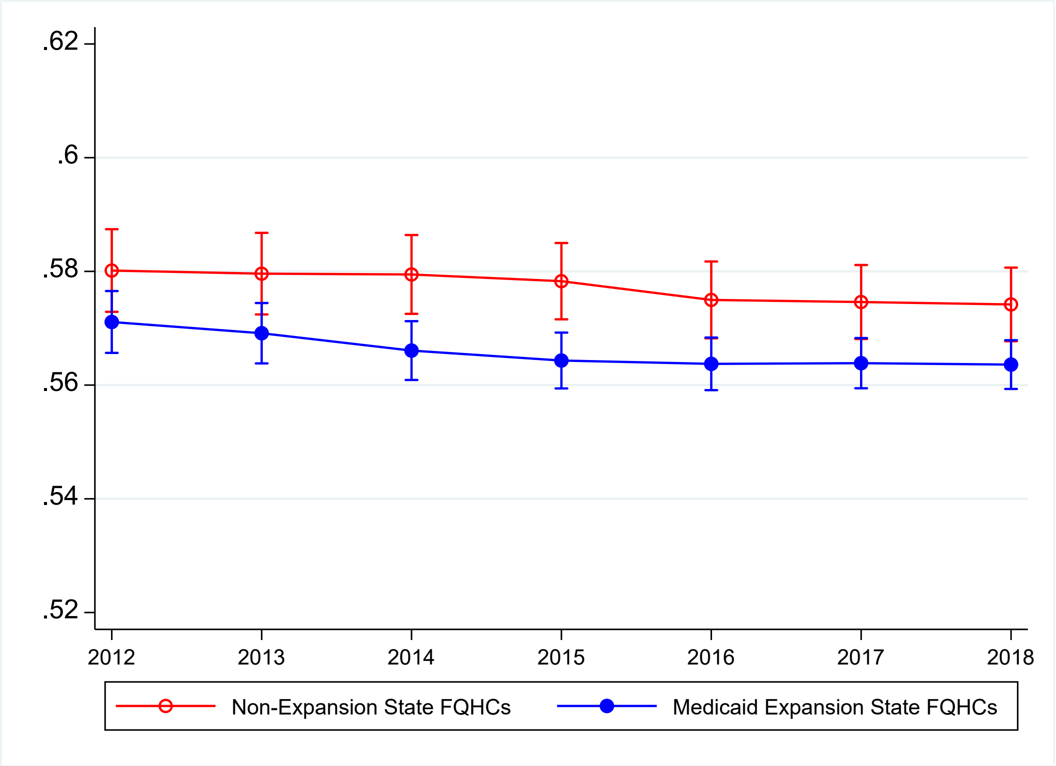

© 2021 Cole MB et al. *JAMA Health Forum.*

eFigure S2-f. Percent of patients under 200% FPL by Medicaid expansion status (2012-2018)

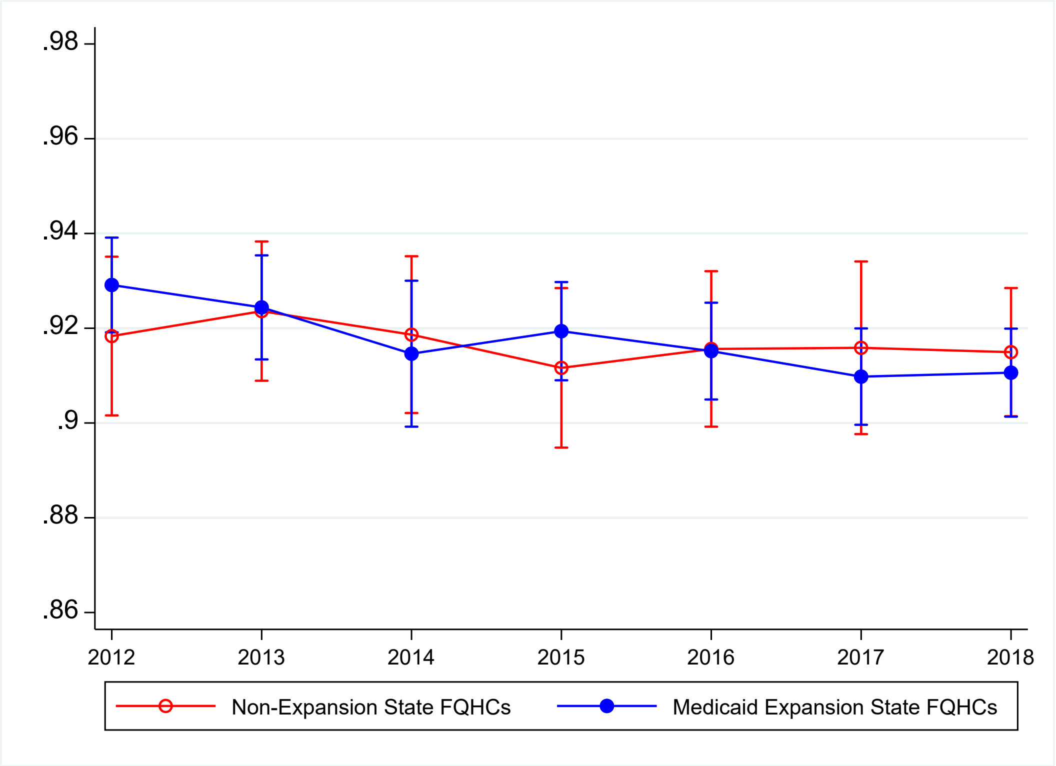

© 2021 Cole MB et al. *JAMA Health Forum.*

eFigure S2-g. Percent of patients who are experiencing homelessness by Medicaid expansion status (2012-2018)

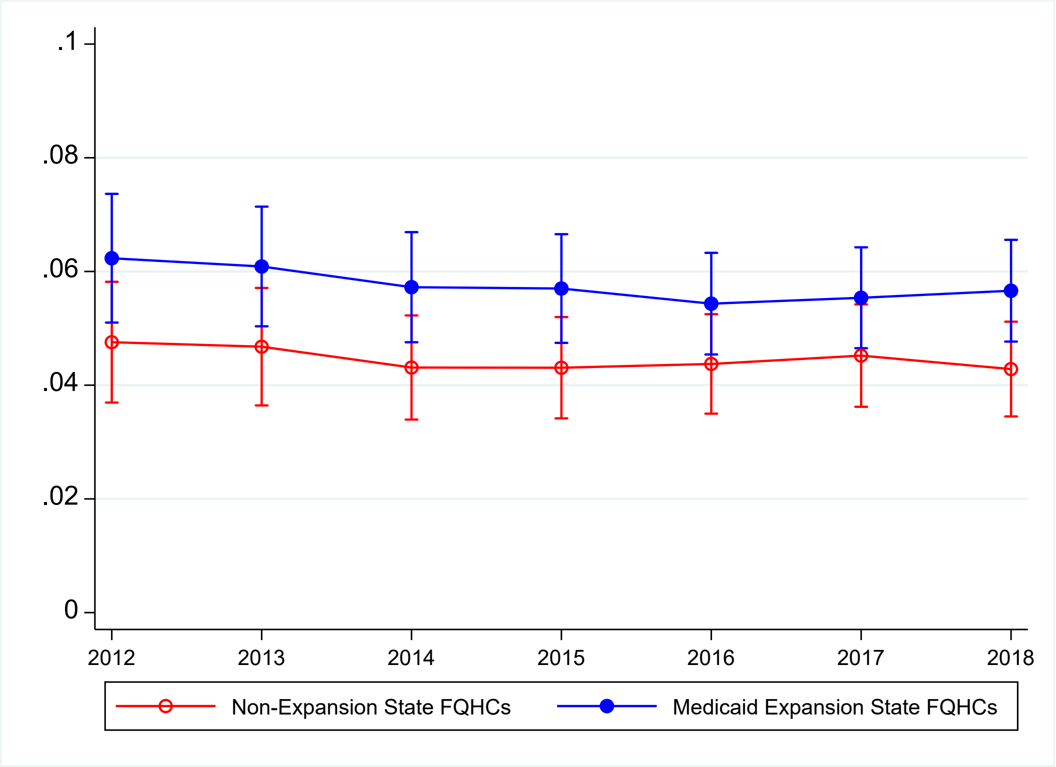

© 2021 Cole MB et al. *JAMA Health Forum.*

eFigure S2-h. Number of patients with hypertension/FQHC by Medicaid expansion status (2012-2018)

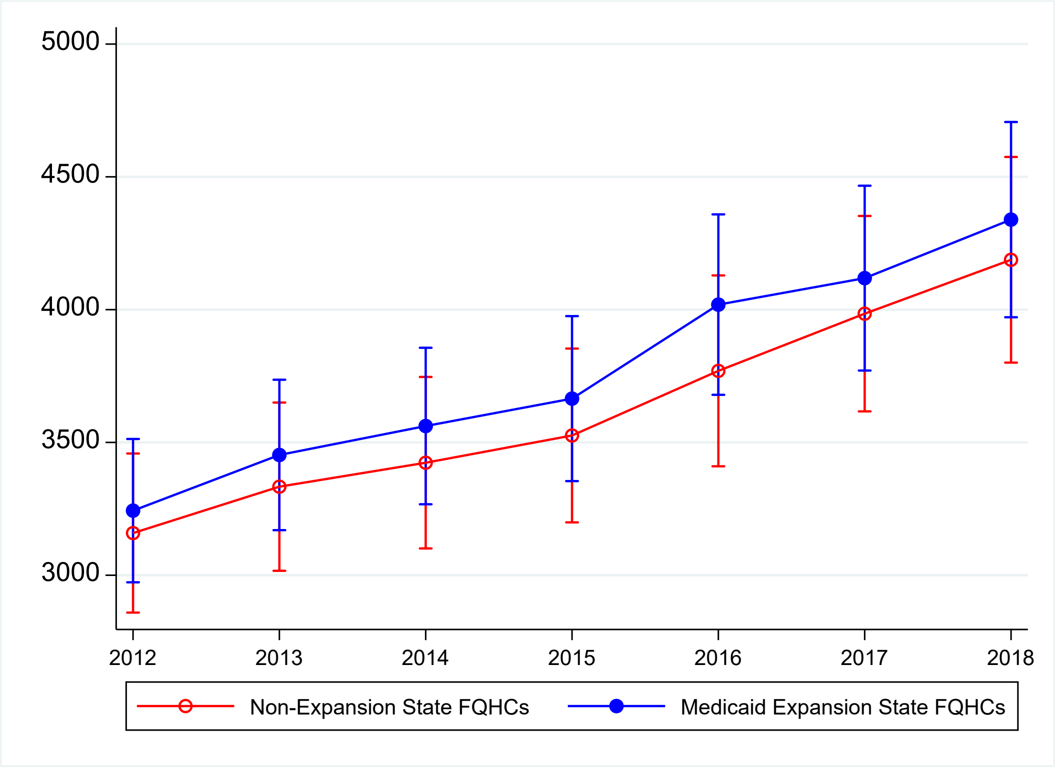

© 2021 Cole MB et al. *JAMA Health Forum.*

eFigure S2-i. Number of patients with diabetes/FQHC by Medicaid expansion status (2012-2018)

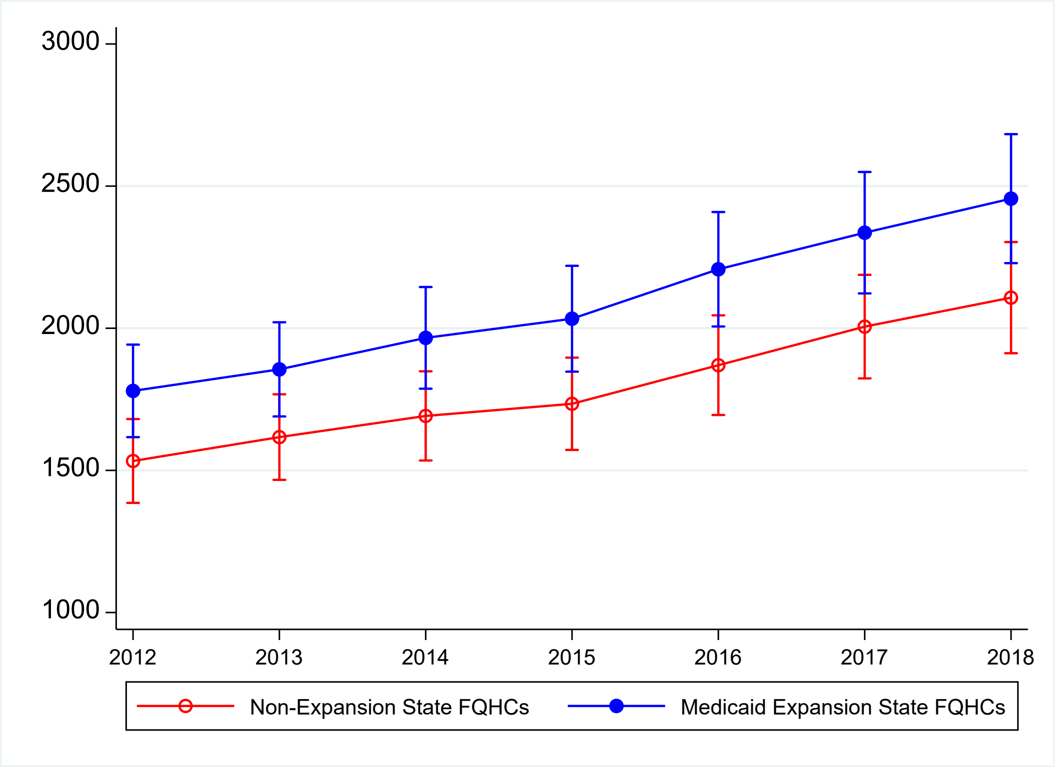

© 2021 Cole MB et al. *JAMA Health Forum.*

E. Changes in insurance coverage: full regression results

eTable 5. Association between Medicaid Expansion and Insurance Coverage in FQHC Adults: Difference-in-Differences Results

	Coef.[a]	Robust SE	t	P>|t|	[95%	CI]
**Uninsured, % adults**						
years 1-5	-9.24	0.66	-13.94	<0.001	-10.54	-7.94
year 1	-8.77	0.59	-14.80	<0.001	-9.93	-7.61
year 2	-9.98	0.71	-14.01	<0.001	-11.38	-8.58
year 3	-9.41	0.75	-12.56	<0.001	-10.88	-7.94
year 4	-9.33	0.78	-11.95	<0.001	-10.86	-7.80
year 5	-8.69	0.79	-10.96	<0.001	-10.25	-7.14
**Medicaid coverage, % adults**						
years 1-5	14.14	0.49	28.61	<0.001	13.17	15.11
year 1	12.66	0.45	28.38	<0.001	11.79	13.54
year 2	15.02	0.54	27.79	<0.001	13.96	16.08
year 3	14.64	0.56	26.10	<0.001	13.54	15.74
year 4	14.45	0.58	25.06	<0.001	13.32	15.58
year 5	13.98	0.59	23.75	<0.001	12.82	15.13

[a] Coefficient represents the adjusted difference-in-difference in the aggregate post period (years 1-5) or in each implementation year, compared to the pre-period (2012-2013). Year 1 is 2014, Year 2 is 2015, Year 3 is 2016, Year 4 is 2017, and Year 5 is 2018.

© 2021 Cole MB et al. *JAMA Health Forum.*

eFigure 3. Association between Medicaid Expansion and Insurance Coverage in FQHC Adults: Difference-in-Differences Results

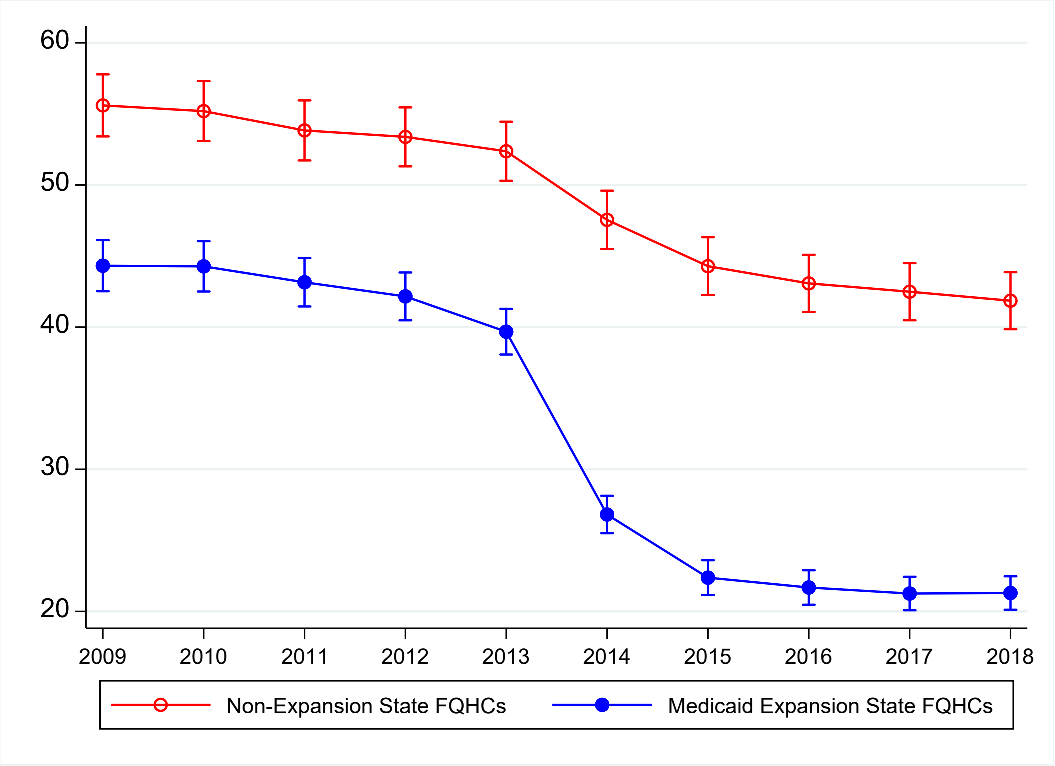

Note: We show trends in uninsurance starting in 2009 in order to illustrate a longer pre-period parallel trend. However, our main manuscript shows results from 2012-2018 only in order to align with the time period of our other analyses, where our health outcome analyses are restricted to 2012-2018 based on available data.

© 2021 Cole MB et al. *JAMA Health Forum.*

F. Sensitivity analyses excluding early expansion states

eTable 6. Association between Medicaid Expansion and Hypertension Control in FQHC Patients: Difference-in-Differences Results – excluding early expansion states[a]

	Coef.[b]	Robust SE	t	P>|t|	[95%	CI]
**All races**						
year 1	1.22	0.61	1.99	0.047	0.01	2.42
year 2	1.15	0.70	1.65	0.100	-0.22	2.52
year 3	1.64	0.73	2.26	0.024	0.21	3.07
year 4	1.76	0.75	2.34	0.020	0.28	3.23
year 5	1.94	0.76	2.55	0.011	0.45	3.44
**White, non-Hispanic**						
year 1	1.47	0.73	2.01	0.045	0.04	2.91
year 2	1.45	0.95	1.52	0.128	-0.42	3.31
year 3	2.38	0.94	2.52	0.012	0.53	4.23
year 4	1.79	0.91	1.97	0.049	0.01	3.57
year 5	1.59	0.92	1.73	0.084	-0.21	3.39
**Black, non-Hispanic**						
year 1	1.60	1.16	1.38	0.167	-0.67	3.87
year 2	1.54	1.39	1.10	0.270	-1.20	4.28
year 3	2.96	1.34	2.22	0.027	0.34	5.58
year 4	2.85	1.50	1.90	0.058	-0.09	5.80
year 5	3.25	1.54	2.11	0.035	0.22	6.28
**Hispanic**						
year 1	0.94	1.24	0.76	0.446	-1.48	3.37
year 2	1.53	1.08	1.42	0.156	-0.58	3.64
year 3	1.19	1.21	0.98	0.326	-1.19	3.57
year 4	2.62	1.15	2.28	0.023	0.36	4.89
year 5	2.76	1.18	2.34	0.020	0.44	5.07
**Asian, non-Hispanic**						
year 1	-2.48	2.56	-0.97	0.332	-7.51	2.54
year 2	-1.97	2.47	-0.80	0.425	-6.81	2.88
year 3	-3.37	2.46	-1.37	0.171	-8.21	1.46
year 4	-2.68	2.49	-1.08	0.282	-7.58	2.21
year 5	-1.97	2.53	-0.78	0.437	-6.93	3.00
**AIAN, non-Hispanic**						
year 1	6.87	5.18	1.33	0.185	-3.29	17.04
year 2	5.47	3.95	1.38	0.167	-2.29	13.23
year 3	8.83	4.61	1.92	0.056	-0.21	17.87
year 4	6.46	4.89	1.32	0.186	-3.13	16.06
year 5	9.20	4.93	1.87	0.062	-0.48	18.87

© 2021 Cole MB et al. *JAMA Health Forum.*

[a] excludes FQHCs in five states (California, Connecticut, Minnesota, New Jersey, and Washington) and the District of Columbia that expanded or partially expanded Medicaid eligibility prior to 2014

[b] Coefficient represents the adjusted difference-in-difference in each implementation year compared to the pre-period (2012-2013). Year 1 is 2014, Year 2 is 2015, Year 3 is 2016, Year 4 is 2017, and Year 5 is 2018.

© 2021 Cole MB et al. *JAMA Health Forum.*

eTable 7. Association between Medicaid Expansion and Diabetes Control in FQHC Patients: Difference-in-Differences Results – excluding early expansion states[a]

	Coef.[b]	Robust SE	t	P>|t|	[95%	CI]
**All races**						
year 1	0.79	0.83	0.96	0.338	-0.83	2.42
year 2	0.40	0.83	0.48	0.631	-1.24	2.04
year 3	2.53	0.87	2.90	0.004	0.82	4.24
year 4	2.48	0.77	3.24	0.001	0.98	3.99
year 5	1.28	0.82	1.57	0.118	-0.33	2.88
**White, non-Hispanic**						
year 1	-0.11	1.07	-0.11	0.916	-2.21	1.98
year 2	-0.28	0.96	-0.29	0.769	-2.16	1.60
year 3	2.67	1.03	2.60	0.009	0.66	4.69
year 4	2.91	0.93	3.14	0.002	1.09	4.73
year 5	0.77	1.01	0.76	0.446	-1.21	2.74
**Black, non-Hispanic**						
year 1	2.84	1.61	1.76	0.079	-0.33	6.00
year 2	4.11	1.58	2.61	0.009	1.02	7.21
year 3	4.43	1.75	2.54	0.011	1.00	7.87
year 4	4.18	1.56	2.68	0.007	1.12	7.23
year 5	3.87	1.67	2.31	0.021	0.58	7.15
**Hispanic**						
year 1	0.80	1.44	0.56	0.576	-2.02	3.62
year 2	-0.47	1.52	-0.31	0.755	-3.45	2.50
year 3	2.90	1.45	1.99	0.047	0.04	5.75
year 4	3.07	1.31	2.35	0.019	0.50	5.65
year 5	2.69	1.35	1.99	0.047	0.03	5.34
**Asian, non-Hispanic**						
year 1	1.36	3.01	0.45	0.652	-4.55	7.27
year 2	-3.95	3.18	-1.24	0.215	-10.18	2.29
year 3	0.07	2.72	0.03	0.978	-5.27	5.42
year 4	0.18	2.87	0.06	0.949	-5.46	5.83
year 5	1.00	3.36	0.30	0.765	-5.59	7.60
**AIAN, non-Hispanic**						
year 1	2.11	4.62	0.46	0.648	-6.96	11.19
year 2	8.80	9.41	0.94	0.350	-9.67	27.27
year 3	-2.20	5.89	-0.37	0.710	-13.77	9.38
year 4	1.78	4.00	0.44	0.657	-6.07	9.63
year 5	1.52	4.62	0.33	0.743	-7.56	10.60

[a] excludes FQHCs in five states (California, Connecticut, Minnesota, New Jersey, and Washington) and the District of Columbia that expanded or partially expanded Medicaid eligibility prior to 2014

[b] Coefficient represents the adjusted difference-in-difference in each implementation year compared to the pre-period (2012-2013). Year 1 is 2014, Year 2 is 2015, Year 3 is 2016, Year 4 is 2017, and Year 5 is 2018.

© 2021 Cole MB et al. *JAMA Health Forum.*

G. Robustness check using placebo post-period

Table S7, below, estimates the difference-in-difference when the pre-period is defined as 2010-2011 and the post-period is defined as 2012-2013. If effects are not null, then our difference-in-differences assumptions may not hold. We do not include 2010-2011 in our analyses due to reporting changes between 2011 and 2012, but use these data here for illustrative purposes only. These results must be interpreted with the caution that the conclusions may not hold if data reporting changes were differential between expansion and non-expansion states.

© 2021 Cole MB et al. *JAMA Health Forum.*

eTable 8. Association between Medicaid Expansion and Intermediate Quality Outcomes in FQHC Patients: Difference-in-Differences Results with Placebo Post Period

**Hypertension Control**						
	Coef.	Robust SE	t	P>|t|	[95%	CI]
All Races	-1.05	0.78	-1.33	0.183	-2.59	0.49
White, non-Hispanic	-0.24	1.17	-0.21	0.837	-2.55	2.06
Black, non-Hispanic	0.29	1.76	0.16	0.870	-3.17	3.74
Hispanic	-1.19	1.85	-0.64	0.520	-4.82	2.44
**Diabetes Control**						
	Coef.	Robust SE	t	P>|t|	[95%	CI]
All Races	-1.38	0.83	-1.66	0.097	-3.00	0.25
White, non-Hispanic	-1.37	1.37	-1.00	0.316	-4.05	1.31
Black, non-Hispanic	-1.56	2.00	-0.78	0.434	-5.49	2.36
Hispanic	-1.65	1.12	-1.47	0.142	-3.86	0.55

© 2021 Cole MB et al. *JAMA Health Forum.*

H. Examination of pre-period parallel trends

eTable 9. Interaction between year and expansion status: 2013 vs. 2012

**Hypertension Control**						
	Coef.	Robust SE	t	P>|t|	[95%	CI]
All Races	-0.09	0.69	-0.13	0.894	-1.44	1.25
White, non-Hispanic	0.17	1.10	0.15	0.880	-1.99	2.32
Black, non-Hispanic	-1.97	1.65	-1.19	0.233	-5.20	1.27
Hispanic	-1.79	1.65	-1.08	0.279	-5.02	1.45
**Diabetes Control**						
	Coef.	Robust SE	t	P>|t|	[95%	CI]
All Races	0.19	0.89	0.22	0.829	-1.56	1.95
White, non-Hispanic	0.31	1.24	0.25	0.800	-2.12	2.74
Black, non-Hispanic	-0.41	1.64	-0.25	0.802	-3.64	2.81
Hispanic	0.99	1.41	0.70	0.486	-1.79	3.76

© 2021 Cole MB et al. *JAMA Health Forum.*

eFigure 4. Percent Patients with Hypertension Control in Pre-Period (all)

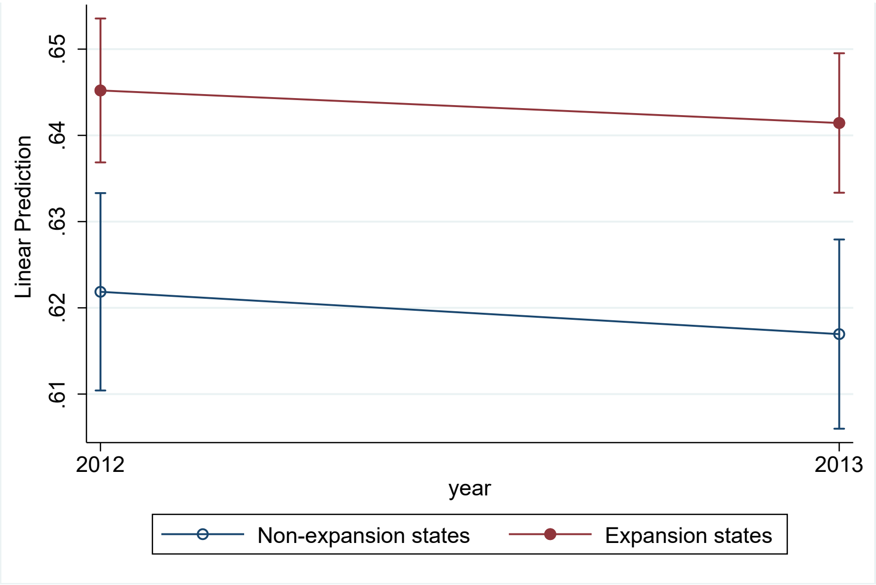

© 2021 Cole MB et al. *JAMA Health Forum.*

eFigure 5. Percent Patients with Diabetes Control in Pre-Period (all)

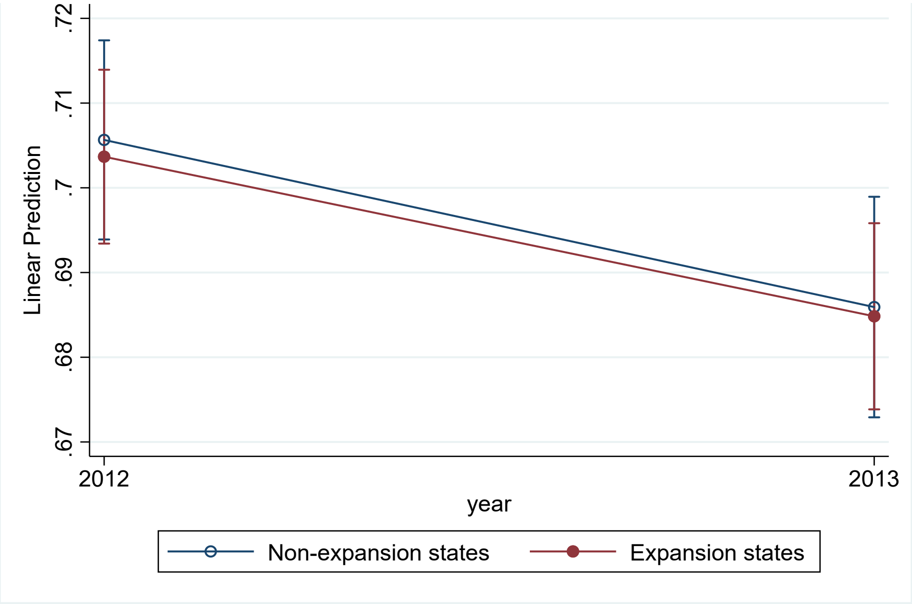

© 2021 Cole MB et al. *JAMA Health Forum.*

eFigure 6. Number of visits for diabetes per FQHC over a longer pre-period (2009-2018)

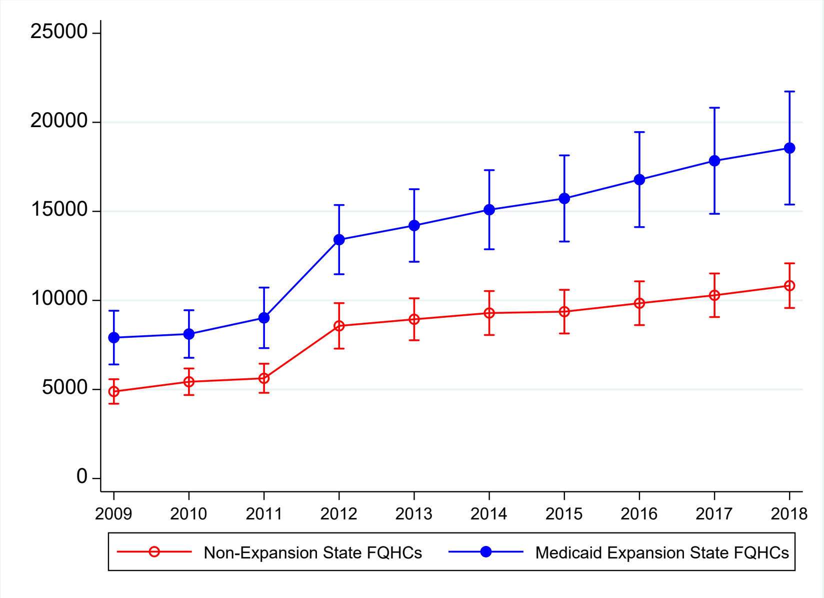

Note: Because our main study outcomes were not reliably available or reported in the UDS prior to 2012, we show pre-period trends in a proxy outcome, number of visits for diabetes/FQHC/year, in this graph. This represents the unique number of visits that had a diagnosis of diabetes.

© 2021 Cole MB et al. *JAMA Health Forum.*

eFigure 7. Number of visits for hypertension per FQHC over a longer pre-period (2009-2018)

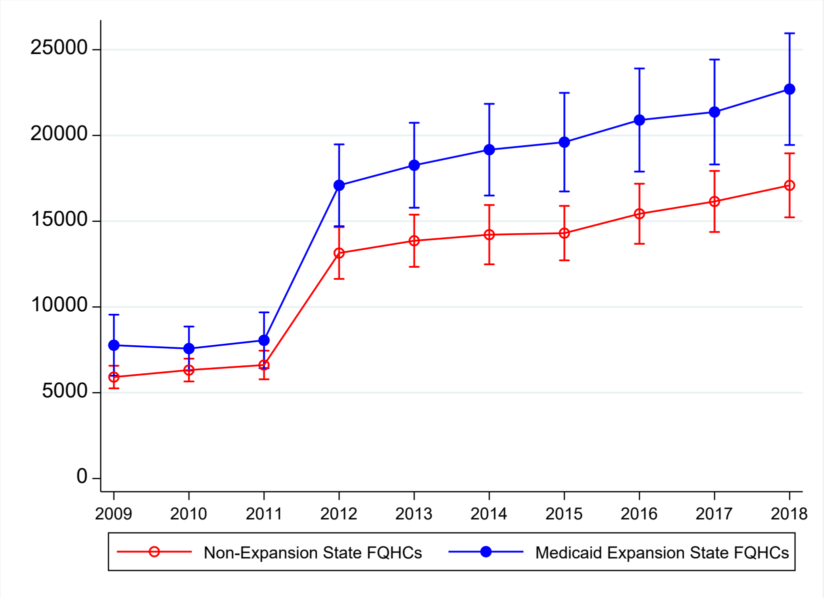

Note: Because our main study outcomes were not reliably available or reported in the UDS prior to 2012, we show pre-period trends in a proxy outcome, number of visits for hypertension/FQHC/year, in this graph. This represents the unique number of visits that had a diagnosis of hypertension.

© 2021 Cole MB et al. *JAMA Health Forum.*

I. Graphs: Hypertension control over time

eFigure 8. Percent FQHC Patients with Hypertension Control by Medicaid Expansion Status (adjusted) – All Races

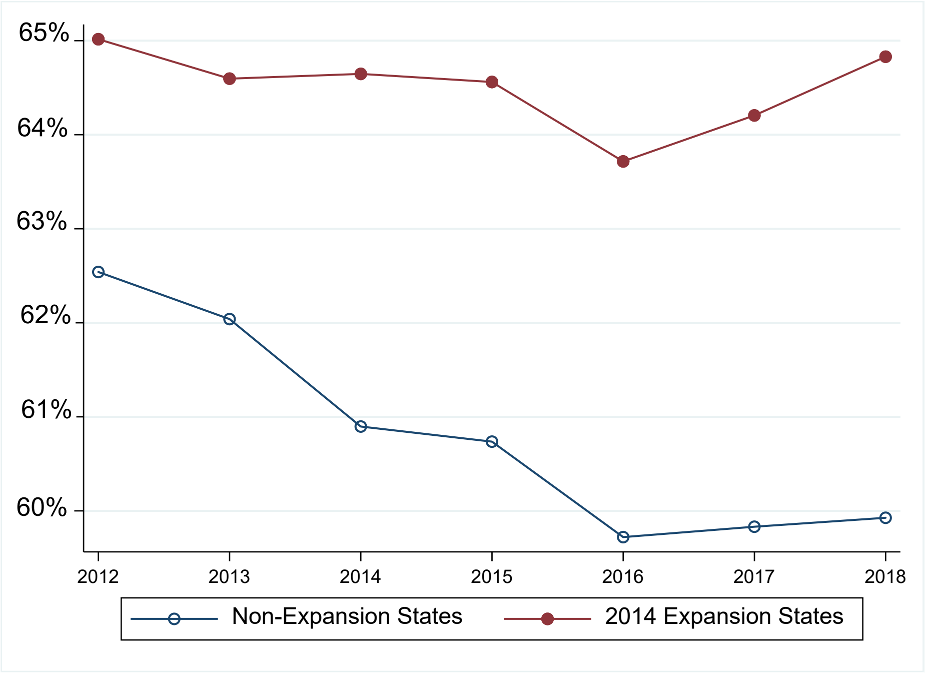

© 2021 Cole MB et al. *JAMA Health Forum.*

eFigure 9. Percent FQHC Patients with Hypertension Control by Medicaid Expansion Status (adjusted) – Black, non-Hispanic

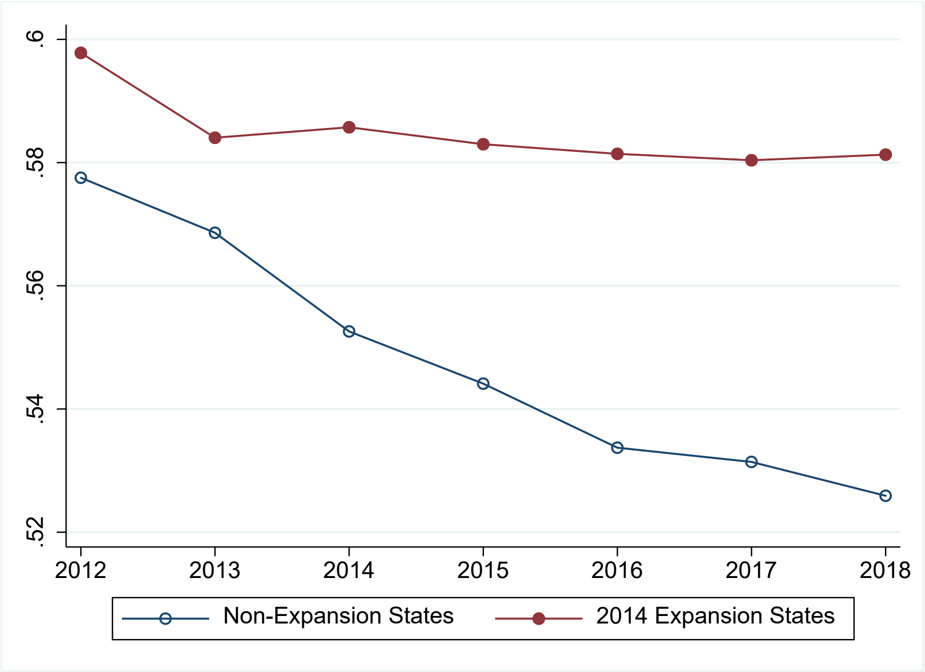

© 2021 Cole MB et al. *JAMA Health Forum.*

eFigure 10 Percent FQHC Patients with Hypertension Control by Medicaid Expansion Status (adjusted) – Hispanic

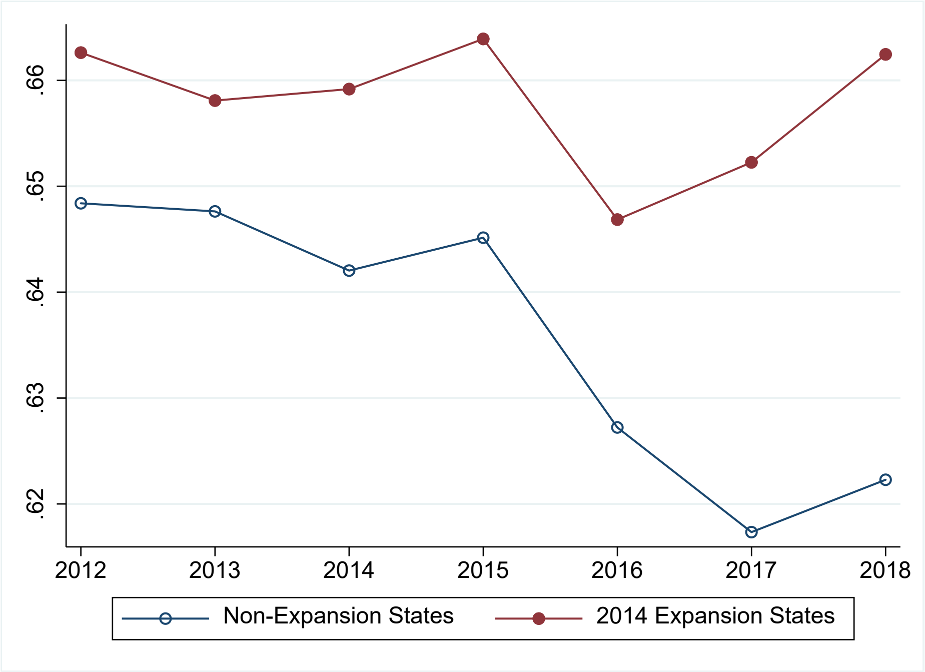

© 2021 Cole MB et al. *JAMA Health Forum.*

J. Graphs: Diabetes control over time

eFigure 11. Percent FQHC Patients with Diabetes Control by Medicaid Expansion Status (adjusted) – all races

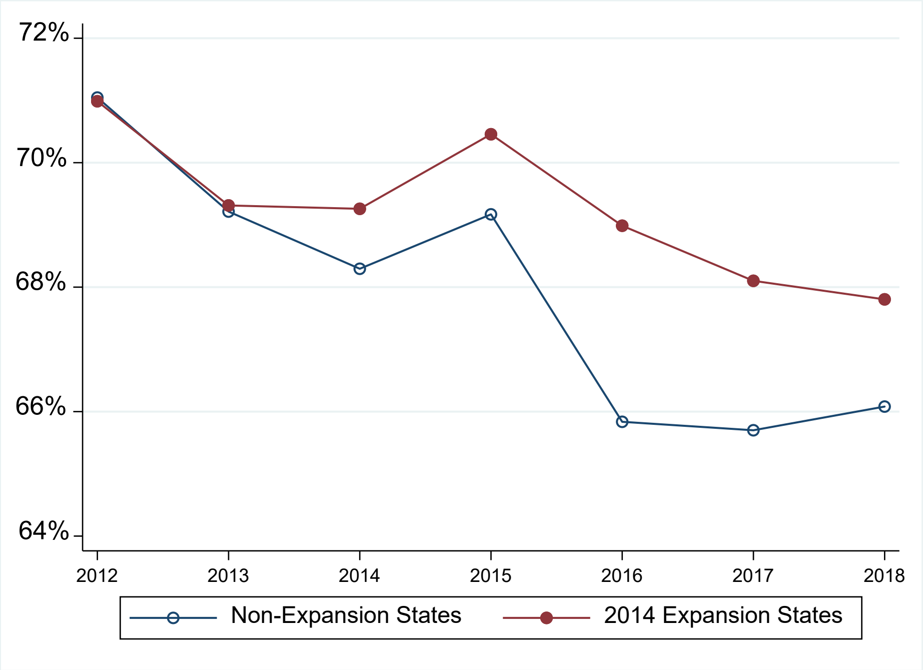

© 2021 Cole MB et al. *JAMA Health Forum.*

eFigure 12. Percent FQHC Patients with Diabetes Control by Medicaid Expansion Status (adjusted) – Black, non-Hispanic

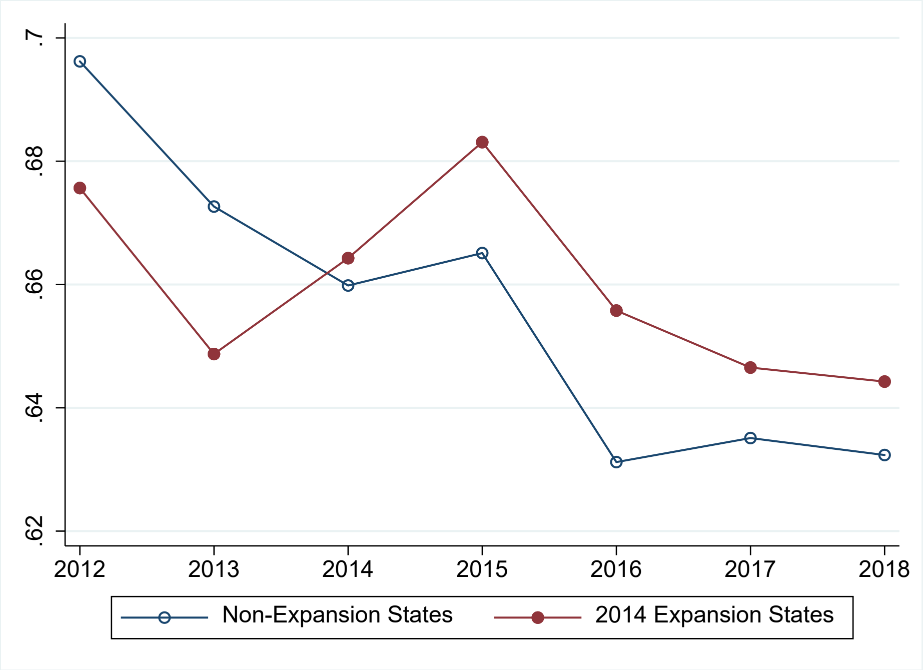

© 2021 Cole MB et al. *JAMA Health Forum.*

eFigure S12. Percent FQHC Patients with Diabetes Control by Medicaid Expansion Status (adjusted) –-Hispanic

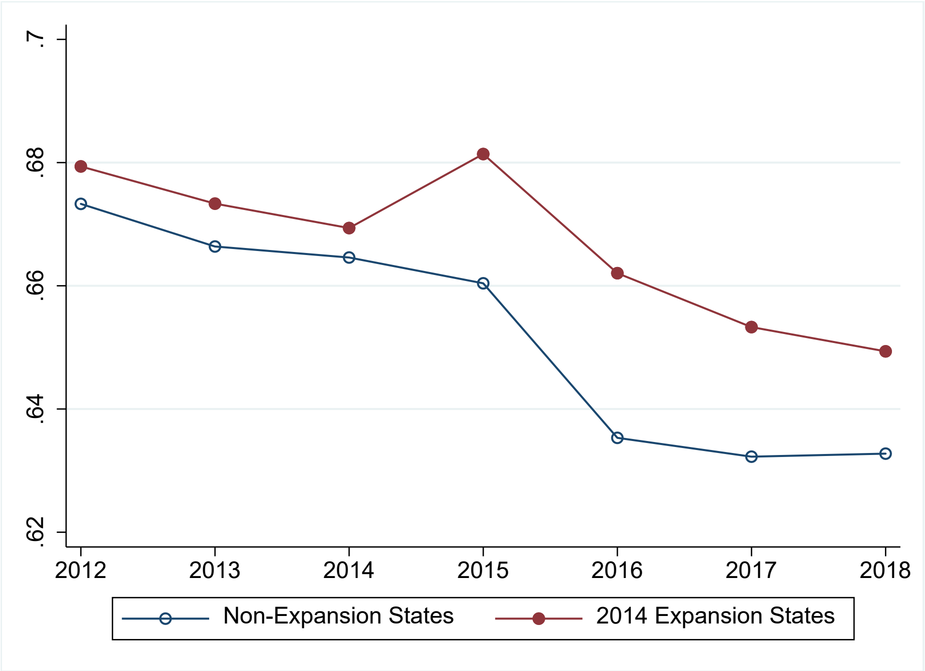

© 2021 Cole MB et al. *JAMA Health Forum.*
